# Molecular Characterization of the Transcription Factors in Susceptible Poplar Infected with Virulent *Melampsora larici-populina*

**DOI:** 10.3390/ijms20194806

**Published:** 2019-09-27

**Authors:** Qiaoli Chen, Jianan Wang, Danlei Li, Zhiying Wang, Feng Wang, Ruizhi Zhang

**Affiliations:** 1 School of Forestry, Northeast Forestry University, Harbin 150040, China; qiaolichen@nefu.edu.cn (Q.C.); wangjianan@nefu.edu.cn (J.W.); fengwang@nefu.edu.cn (F.W.); zhangruizhi1993@163.com (R.Z.); 2 Key Laboratory of Sustainable Forest Ecosystem Management-Ministry of Education, Northeast Forestry University, Harbin 150040, China

**Keywords:** *Melampsora larici-populina*, *Populus*, transcription factors, susceptibility, resistance, compatible interaction

## Abstract

Transcription factors (TFs) have been shown to play important roles in determining poplar susceptibility. In this study, the transcript profiles of five resistance-related TF groups at different time points were investigated to study the roles of TFs in the compatible interaction between ‘Robusta’ (*Populus nigra* × *P. deltoides*) and the virulent E4 race of *Melampsora larici-populina*. The susceptibility test indicated that the parasitic process of E4 could be divided into two representative time periods: the infection phase and the production phase. Bioinformatics analysis showed that in these two phases, E4 infection induced a network of TFs in ‘Robusta’. Although some TFs responded rapidly and positively, most TFs did not respond to E4, especially during the infection phase. The ethylene, jasmonic acid, and auxin pathways were downregulated, while a calcium-binding protein was upregulated. No other significantly changed phytohormone-related genes were found, which was consistent with the pathological process in the absence of an immune response, suggesting that the lack of response of most TFs during the infection phase of E4 is related to the susceptibility of ‘Robusta’.

## 1. Introduction

Plants utilize an intricate immune system that responds to microbial pathogen infections [1]. First, the recognition of conserved pathogen-associated molecular patterns (PAMPs) by cell-surface pattern-recognition receptors (PRRs) results in PAMP-triggered immunity (PTI) [1,2,3,4]. Receptor-like kinases (RLKs, also known as receptor kinases) and receptor-like proteins (RLPs) have been identified to function as PRRs [5]. The relaying of PTI signaling involves the association and activation of receptor-like cytoplasmic kinases (RLCKs) with PRR complexes. Upon the perception of PAMPs by PRR complexes [6,7,8,9], immune signaling is transduced through the mitogen-activated protein kinase (MAPK) cascade [10,11] and calcium-dependent protein kinases (CDPKs) [12], resulting in a series of defensive responses, such as reactive oxygen species (ROS) burst, stomata closure, the initiation of the salicylic acid (SA), and jasmonic acid (JA) signaling pathways, and the expression of immune-related genes. Such defense responses can limit the colonization by infectious pathogens [13]. To counteract PTI, pathogens have evolved effector molecules to attenuate PTI [1]. An additional form of induced defense is triggered in response to the recognition of pathogen effectors and is called effector-triggered immunity (ETI) or R-mediated resistance [14].

Innate immunity is regulated at different levels to ensure the optimal intensity and duration of immune responses. In plant immune signaling pathways, MAPK and CDPK can regulate the expression of plant immune-related genes by phosphorylating downstream transcription factors (TFs). Many TFs are key participants in plant immune responses [15] as they can regulate plant immunity by regulating the expression of downstream genes [16]. TF ERF6 of the ERF family can be phosphorylated by MPK3/MPK6. Phosphorylated ERF6 can activate the expression of immune-related genes in plants, making them more resistant to pathogens [17]. Based on the spatial structure of proteins, 41 of the 131 members of the MYB family might interact with MAPK3 [18]. When the MAPK signaling pathway was activated, the MYB family TF MYB44 was phosphorylated by MPK3 to activate its transcriptional activity and regulate the transcription of plant immune-related genes [19]. Members of the *WRKY* gene family are also widely involved in plant immunity [20]. For example, WRKY22 and WRKY29 are activated by MPK3/6 and play a positive regulatory role [21]. WRKY1 of tobacco (*Nicotiana tabacum*) can be phosphorylated by the MAP kinase salicylic acid-induced protein kinase (SIPK) and can mediate hypersensitive response (HR)-like cell death [22]. Similarly, SIPK can also catalyze the phosphorylation of WRKY8 at multiple sites, thus enhancing the expression of downstream immune genes [23]. Furthermore, TFs also participate in regulating the cross-talk between different defensive signaling pathways [16]. Several small messenger molecules are involved in translating the pathogen-induced early signaling events into activation of effective defense responses, such as SA, JA, and ethylene (ET) [24,25]. Some TFs are important nodes of convergence of phytohormone signaling and play important roles in the regulation of phytohormone-responsive genes [26].

The plant PTI defense system is a defense mechanism that can induce resistance with diverse adaptability and is characterized by broad-spectrum resistance. Each pathogen has PAMPs that induce PTI when it infects plants, but some pathogens can overcome PTI in plants by their effector molecules. Additionally, if ETI is absent, the pathogen will multiply in the plant and make the plant susceptible. The hybrid poplar ‘Robusta’ (*Populus nigra* × *P. deltoides*) is susceptible to the virulent E4 race of *Melampsora larici-populina* [27]. A previous study indicated that ‘Robusta’ shows nonrace-specific resistance to the E1, E2 and E3 races of *M. larici-populina* and sensitivity to the new E4 race of *M. larici-populina*. It has been reported that the transcript levels of TFs, such as AP2/ERFs, MYBs and WRKYs, were altered in ‘Robusta’ after infection with E4 [27]. Our studies on TFs aimed to elucidate the transmission process of disease-resistance signals and the mechanisms that enable E4 to overcome the PTI of the susceptible poplar, which may provide a better understanding of the susceptibility of compatible hosts to biotrophic pathogens.

## 2. Results

### 2.1. In Vivo Monitoring of E4 Growth

**Figure 1:**
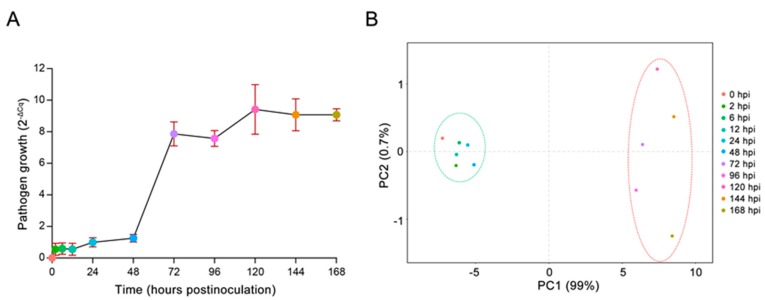
E4 growth curves and the PCA assessment. (**A**) E4 growth curves were determined by qPCR. (**B**) The PCA assessment of E4 DNA mass covariance showed that the 11 time points could be divided into two main groups.

‘Robusta’ was clearly susceptible to E4, as the disease score was 4 (slope factor = 1.328, average maximum slope factor (AMSF) = 1.076). Additionally, the reproduction of E4 at 11 time points was examined by qPCR (Figure 1A). After inoculation on ‘Robusta’, the DNA mass of E4 increased sharply from 2 dpi to 3 dpi. The principal component analysis (PCA) assessing the DNA mass covariance of E4 showed that those 11 time points could be divided into two main groups (Figure 1B). Based on these results, samples of different time points could be clustered into two groups to allow a relevant statistical analysis: the infection phase (0 hpi to 2 dpi) and the production phase (3 dpi to 7 dpi). According to the pathological process [28], the period during which the infection hyphae extended into the mesophyll was 12 hpi (Figure S1A). A dense network of infection hyphae and haustoria formed in the mesophyll near the primary infection site at 4 dpi (Figure S1B) [29]. Hence, 12 hpi and 4 dpi were selected as representative time points for the infection phase and the production phase, respectively.

### 2.2. Identification of Differentially Expressed Poplar Genes Following E4 Infection via Digital Gene Expression Sequencing

In this study, differentially expressed genes (DEGs) were defined by default as those with false discovery rate (FDR) ≤ 0.05 and multiple differences of more than 2 times (log_2_fold change > 1 or < −1). In total, 14,008 DEGs were identified in the 12 hpi library and 15,888 DEGs were identified in the 4 dpi library. Among all of these DEGs, 8507 genes were identified as DEGs in both libraries (co-DEGs, Figure S2). Among these co-DEGs, 1228 were identified as significant DEGs (FDR ≤ 0.01, Table S1).

### 2.3. Infection with E4 Induced a Network Located in the Nucleus

**Figure 2:**
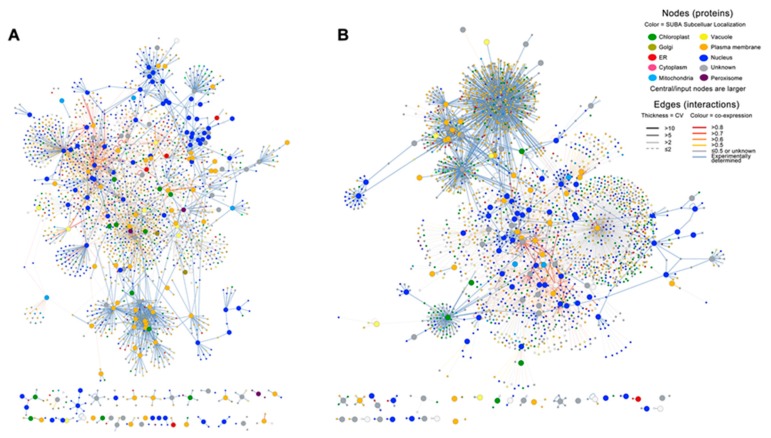
The subcellular location of E4 response ‘Robusta’ proteins. (**A**) E4 response proteins of ‘Robusta’ encoded by upregulated genes. (**B**) E4 response proteins of ‘Robusta’ encoded by downregulated genes. The prediction was based on the subcellular location database for *Arabidopsis* (SUBA; http://suba.plantenergy.uwa.edu.au). These results indicate that the top group of those proteins were located in the nucleus.

The interactions and subcellular localizations of proteins encoded by 1228 significant DEGs were predicted based on the SUBcellular location database for *Arabidopsis* (SUBA, http://suba.plantenergy.uwa.edu.au). There were 1008 homologous proteins (575 for co-upregulated DEGs, Figure 2A; 433 for co-downregulated DEGs, Figure 2B) and 6922 interactions (3311 interactions, Figure 2A; 3611 interactions, Figure 2B) identified in *Arabidopsis*. These proteins clustered into two major subcellular locations. The first group was located in the nucleus (encoding gene expression levels where 46.13% of them were upregulated and 20.65% of them were downregulated), the second group was located in the plasma membrane. This suggests that the infection with E4 induced a network located in the nucleus.

### 2.4. GO Category Analysis Illustrated an Enrichment of Genes Associated with TFs

Gene Ontology (GO) analysis was performed for the 8507 co-DEGs responding to E4. Altogether, 2781 genes were annotated in GO terms. Among them, 281 were enriched in DNA-dependent transcription regulation (GO: 0006355, *p*-value = 1.00 × 10^−7^, FDR = 1.60 × 10^−5^, Figure S3A). Upon further enrichment of those 281 genes, 138 were enriched in TF activity (GO: 0003700, *p*-value = 1.20 × 10^−116^, FDR = 4.30 × 10^−115^, Figure S3A), including processes related to transcription regulator activity (GO: 0030528, 173 genes, *p*-value = 2.10 × 10^−141^, FDR = 1.40 × 10^−139^), sequence-specific DNA binding (GO: 0043565, 84 genes, *p*-value = 6.90 × 10^−62^, FDR = 1.90 × 10^−60^) and protein dimerization activity (GO: 0046983, 34 genes, *p*-value = 3.10 × 10^−21^, FDR = 6.20 × 10^−20^). This result indicated that TF genes were activated. The GO enrichment analysis indicated that the differentially expressed poplar genes were associated with 20 terms within the biological processes category, such as the response to biotic stimulus (GO: 0009607, *p*-value = 1.1 × 10^−4^, FDR = 9 × 10^−3^) and the metabolic process (GO: 0008152, *p*-value = 2.10 × 10^−78^, FDR = 1.00 × 10^−77^, Figure S3B).

### 2.5. E4 Infection Did Not Affect Most TFs

**Figure 3:**
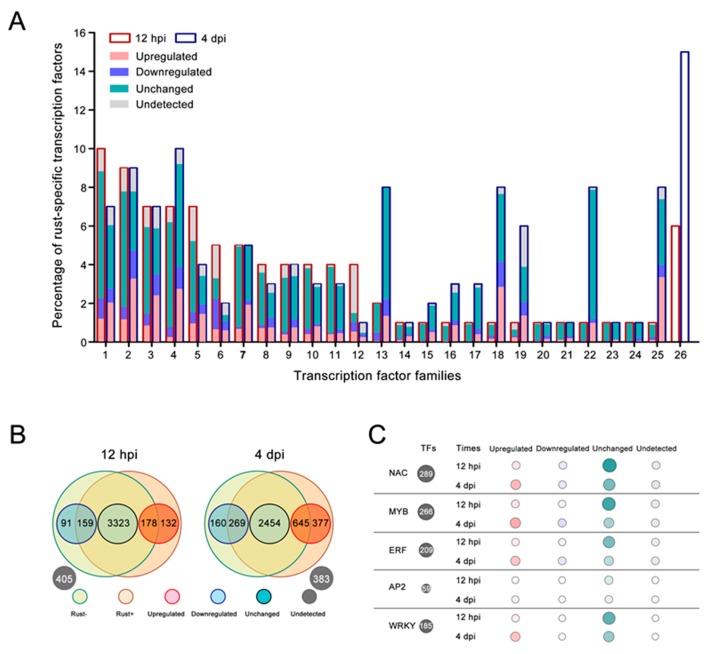
Transcriptional changes in the transcription factor (TF) family genes in ‘Robusta’ following infection with E4. (**A**) Proportion of different TF family genes after E4 infection. 1: *NAC*, 2: *MYB*, 3: *ERF*, 4: *bHLH*, 5: *B3*, 6: *LBD*, 7: *WRKY*, 8: *MIKC*, 9: *C2H2*, 10: *G2-like*, 11: *MYB_related*, 12: *M-type*, 13: *HSF*, 14: *AP2*, 15: *HD-ZIP*, 16: *GRAS*, 17: *bZIP*, 18: *CPP*, 19: *WOX*, 20: *Dof*, 21: *SBP*, 22: *Trihelix*, 23: *FAR1*, 24: *C3H*, 25: *GRF*, and 26: others. (**B**) Summary of differentially expressed TF genes. Rust-: E4 free ‘Robusta’; rust+: E4 infected ‘Robusta’; upregulated: log_2_(rust+/rust-) ≥ 1; downregulated: log_2_(rust+/rust-) ≤ −1; unchanged: −1 < log_2_(rust+/rust-) <1. (**C**) The *NAC*, *MYB*, *ERF*, *AP2*, and *WRKY* genes induced by E4 infection in ‘Robusta’. The size and color of the bubble represent the gene number in each TF family. A larger size or darker color indicates the presence of more genes (detailed in Table S2).

Most TFs did not respond to the virulent E4 race, particularly at 12 hpi, when only 13% of the TFs of ‘Robusta’ were responsive to E4. However, 33.8% of TFs were induced by E4 at 4 dpi. A total of 4288 TFs from 58 families were analyzed based on DGE sequencing results (Table S2). Altogether, 310 TF genes were upregulated, 250 TF genes were downregulated, 3323 TF genes were unchanged, and another 405 TF genes were undetectable in both the rust- and rust+ libraries at 12 hpi (Table S2). In addition, 1022 upregulated, 429 downregulated and 2,454 unchanged TF genes were identified at 4 dpi, with another 383 TF genes undetectable in both the rust- and rust+ libraries (Table S2). The genes from 25 TF families that accounted for the vast majority of all TFs at 12 hpi were listed and compared with those from 4 dpi (Figure 3A). Among all upregulated TF genes, 132 and 377 TFs specifically belonged to rust+ libraries at 12 hpi and 4 dpi, respectively (Figure 3B). Additionally, the expression changes in resistance-related TFs (NAC, MYB, ERF, AP2, and WRKY) are listed in Figure 3C. Notably, there were more unchanged resistance-related TF genes at 12 hpi and more upregulated resistance-related TF genes at 4 hpi.

### 2.6. Resistance-Related TF Families Induced by E4 in ‘Robusta’

**Figure 4:**
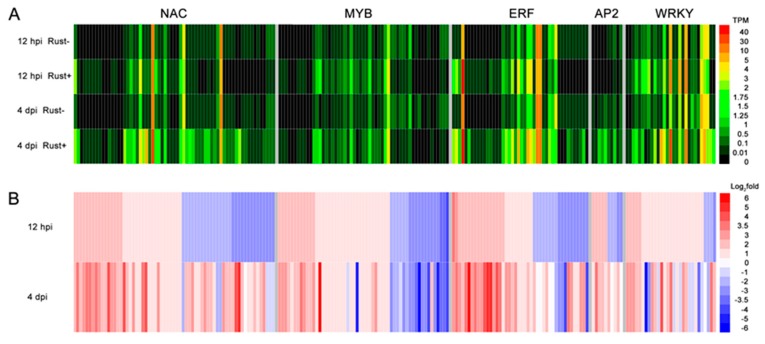
Expression profile of 5 resistance-related TF families. (**A**) Transcripts per million -normalized reads for 5 TF families from E4 uninfected (rust-) and infected (rust+) sequencing data at 12 hpi and 4 dpi. These results indicated that the rust fungal infection could not only induce, but also suppress TF genes. The color code axis represents normalized TPM. (**B**) Expression changes in 5 TF families following infection with E4 at 12 hpi and 4 dpi. The color code axis represents log_2_(rust+/rust-) fold changes in TPM.

To obtain additional detailed information, the expression profiles of *NAC*, *MYB*, *ERF*, *AP2* and *WRKY* were generated according to the TPM-normalized DGE sequencing reads (Figure 4A). Many TFs were undetectable in rust- libraries but could be detected in rust+ libraries. However, many detectable TFs were undetected at 12 hpi. These results indicated that E4 infection could not only induce, but also suppress the expression of TF genes. Altogether, the expression of 14 *NAC* genes, 10 *MYB* genes, 12 *ERF* genes, 5 *AP2* genes and 2 *WRKY* genes was induced, and the expression of 11 *NAC* genes, 9 *MYB* genes, 8 *ERF* genes, 1 *AP2* gene and 1 *WRKY* gene was suppressed at 12 hpi (Figure 4A). Furthermore, many upregulated/downregulated TF genes were downregulated/upregulated when E4 infected ‘Robusta’ (from 12 hpi to 4 dpi, Figure 4B). These results suggested that TF genes have complex expression profiles and that the DGE data from two time points could not reveal the entire expression patterns of the TF genes.

### 2.7. TF-Binding Motifs Show Specific Enrichment in DEG Groups

**Figure 5:**
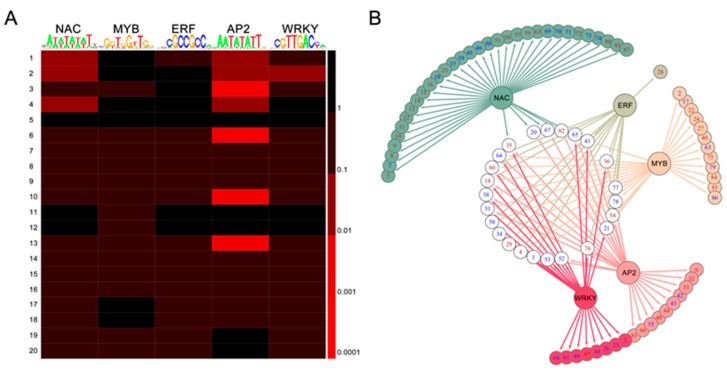
The overrepresentation of 5 TF-binding motifs in the promoters of DEGs. (**A**) Five TF-binding motifs are differentially enriched in the promoters of differentially expressed (promoted and suppressed) genes during E4 infection. The scale corresponds to raw p-values. (**B**) The network of 5 TF families regulates potential target genes with the corresponding TF-binding motif, detailed in Table S3. Red and blue labels represent upregulation and downregulation, respectively.

The GO enrichment of DEGs identified clusters of genes that participated in the same physiological process and, thus, may be coregulated. To gain an initial understanding of the poplar susceptibility regulatory mechanisms related to TFs during E4 infection, promoters corresponding to the region 500 bp upstream of the predicted transcription start sites of genes in each GO cluster were screened for overrepresentation of *NAC*-, *MYB*-, *ERF*-, *AP2*-, and *WRKY*-binding motifs based on the genomic data of *P. trichocarpa* V 3.0 (Figure 5A). Then, a predicted potential target gene regulatory network was generated (Figure 5B, Table S3). The results indicated that most potential target genes were regulated by more than one TF, and their target genes were also dependent on several TFs.

The global maps indicated that 147 Kyoto Encyclopedia of Genes and Genomes (KEGG) pathways were related to potential TF target genes after infection with E4 (Table S4). Altogether, 20 metabolic pathways were upregulated (Figure S4, red line) or downregulated (Figure S4, blue line). No significantly changed gene was found, except for the 3 phytohormone signal transduction pathways (the ET, JA, and auxin pathways), which were downregulated, and a calcium-binding protein related to the HR, which was upregulated. Pathways of tropane, flavonoid biosynthesis, piperidine and pyridine alkaloid biosynthesis, ubiquinone and other terpenoid-quinone biosynthesis, carbon fixation in photosynthetic organisms, monoterpenoid biosynthesis, ascorbate metabolism, and aldarate metabolism were upregulated, while pathways of glycolysis/gluconeogenesis, fatty acid biosynthesis and degradation, degradation of aromatic compounds, cytochrome P450, N-glycan biosynthesis, and photosynthesis were downregulated. In addition, some pathways were disarranged (some genes were upregulated and some were downregulated in the same pathway), such as the pathways of phenylpropanoid biosynthesis, carbohydrate metabolism, biosynthesis of amino acids, and metabolism of terpenoids and polyketides.

### 2.8. NAC, MYB, ERF, AP2, and WRKY Genes Did Not Respond to E4 during the Infection Phase

**Figure 6:**
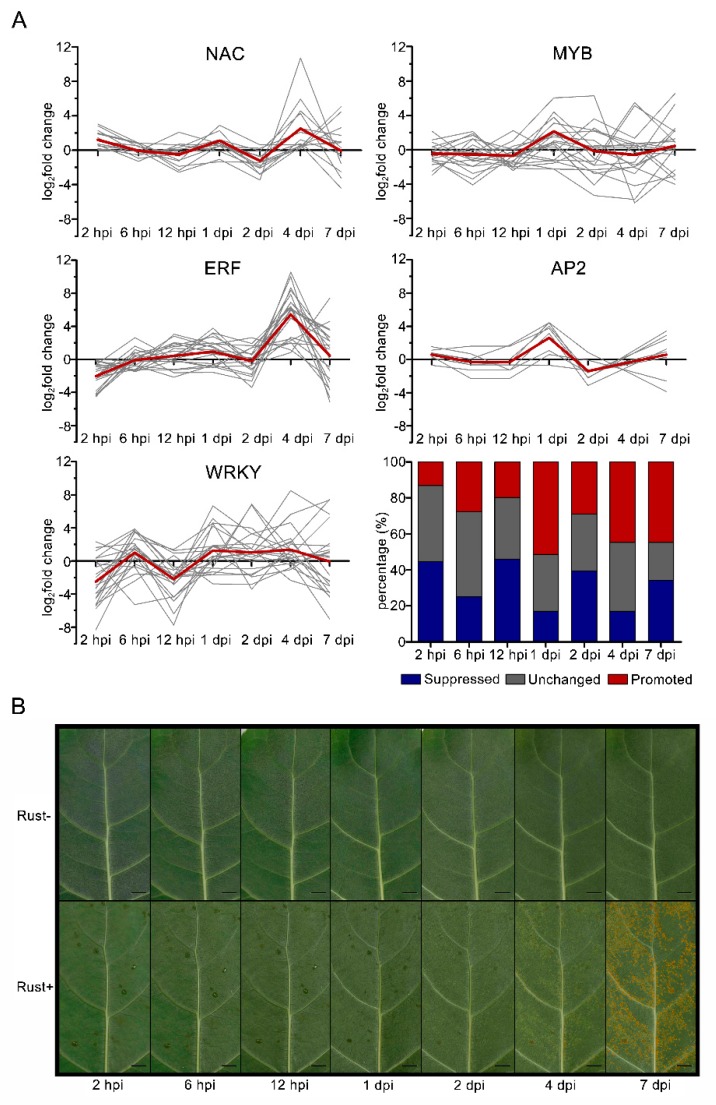
Cluster analysis for expression levels of 5 TF families and symptoms at different time points post inoculation. (**A**) Cluster analysis of TF expression levels at different time points by RT-qPCR. Almost all of the *NAC*, *MYB*, *ERF*, *AP2*, and *WRKY* genes were promoted at 1 dpi. (**B**) Symptoms of ‘Robusta’ at different time points post inoculation. The scale bars represent 1 mm.

RT-qPCR was performed for the *NAC*, *MYB*, *ERF*, *AP2*, and *WRKY* genes at 2 hpi, 6 hpi, 12 hpi, 1 dpi, 2 dpi, 4 dpi, and 7 dpi to obtain detailed expression information at different time points (Figure 6A). Collectively, the RT-qPCR results showed that the changes in the expression of the TFs had no obvious regularity. However, the signatures of many TF families indicated that their process from the infection phase to the production phase was similar. The *NAC* and *ERF* genes were activated at 1 dpi and then reached a peak at 4 dpi. The expression of *MYB* genes showed variation from the infection phase to the production phase, but many of them peaked at 1 dpi. The *AP2* genes had only one fluctuation, which also peaked at 1 dpi. The *WRKY* genes were activated at 6 hpi first and then activated at both 1 dpi and 4 dpi. Our results indicated that there were more downregulated TF genes at the infection phase than at the production phase, and more upregulated TF genes at the production phase than at the infection phase, especially for *WRKY* genes. The RT-qPCR results of each TF family were clustered, and the results indicated that most of the *NAC*, *MYB*, *ERF*, *AP2*, and *WRKY* genes were unchanged or downregulated from 2 hpi to 12 hpi. These results indicated that those 5 TF family genes did not respond to E4 rust during the infection phase.

## 3. Discussion

TFs are proteins that control target gene expression levels and modulate rates of transcription. The functional characterization of TFs is expanding at an increasing pace, but there are still few examples available for poplar [30]. The previous DGE library sequencing, which was based on a mixture of samples harvested at different time points, identified TFs that were downregulated or upregulated following the infection of the virulent E4 race of *M. larici-populina* [27]. To avoid overlooking differences in the mixture of samples, DGE sequencing at two time points and RT-qPCR at 7 time points were performed in this study. The results suggested that the infection of E4 induced a gene network of TFs. Potential target genes were regulated by more than one TF, and their target genes were also dependent on several TFs. No hypersensitive response (HR) symptoms were detected (Figure 6B). Only one HR-related gene, a calcium-binding protein gene, was upregulated after infection with E4. Most importantly, three phytohormone signal transduction pathways were downregulated in response to E4 infection.

There is a compatible interaction between ‘Robusta’ and E4 [27]. A previous study indicated that ‘Robusta’ shows nonrace-specific resistance to the E1, E2, and E3 races of *M. larici-populina* but sensitivity to the new E4 race of *M. larici-populina*. It is obvious that there is a gap between the altered metabolism of ‘Robusta’ and the pathological process of *M. larici-populina* during their evolutionary process. However, the main factor that enabled E4 to overcome ‘Robusta’ resistance remains unknown. Because phenotype is closely related to metabolism, the TF changes affecting metabolism were studied based on the KEGG pathways. The global maps indicated that after ‘Robusta’ was infected by E4, 147 KEGG pathways were observed to be related to potential TF target genes (Table S4).

Both PTI and ETI lead to the activation of various signaling transduction pathways involving MAPK, ROS, SA, JA, ET, and other phytohormones and signaling molecules, which eventually result in the production of antimicrobial compounds and secondary metabolites, the modification of cellular structure through callose deposition, and programmed cell death (PCD) [10,31]. These are achieved by transcriptional regulators forming an important node to balance growth and defense for the optimal allocation of resources and survival of plants [32]. Although some TFs responded rapidly and positively, our results also revealed that most TFs of ‘Robusta’ were not affected by E4 at the infection phase, while many TF genes were upregulated at the production phase. However, most TFs of ‘Robusta’ were not affected by E4, particularly many resistance-related TFs [31], such as NAC, MYB, ERF, AP2 and WRKY. These TFs mostly failed to respond during the infection phase. The ET, JA, and auxin pathways were downregulated, a calcium-binding protein was upregulated, and no other significantly changed phytohormone-related genes were found. These results were consistent with the pathological process in the absence of an immune response (Figure 6B).

Most of the *NACs* and many *MYBs* and *AP2s* were significantly up-regulated at 2 hpi, indicating that these three resistance-related TF families may respond positively to E4 infection at the very beginning. We also observed that most *WRKY*s and *ERF*s were obviously downregulated at 2 hpi (Figure 6A). ERFs constitute the largest family of TFs in *Arabidopsis*, with more than 120 members. Many ERFs have been implicated in plant defense responses [17]. WRKYs are a large family involved in various plant processes, but most notably involved in coping with diverse biotic and abiotic stresses [20]. The results indicated that infection with E4 may have a substantial impact on these two TF families, and that these two TF families may play an important regulatory role in the early stage of infection. Many *NAC*s, *ERF*s, and *WRKY*s were upregulated at 4 dpi, when a dense network of infection hyphae and haustoria forms in the mesophyll near primary infection sites, indicating that infection with E4 may cause further reactions in ‘Robusta’. However, this late reaction did not prevent the infection of E4. Considering these results, we deduced that the susceptibility of ‘Robusta’ can be related to the lack of response of most TFs at the infection phase of E4.

## 4. Materials and Methods

### 4.1. Rust Isolation, Plant Materials, and Inoculation Procedure

The filial generation of virulent E4 was collected from *P. trichocarpa* cv. Trichobel in Markington (Northern England) according to previously reported methods [33]. One-year-old hybrid poplar ‘Robusta’ (*P. deltoides* × *P. nigra*) was grown and inoculated as described by Pei et al. [33]. A spore suspension of E4 was adjusted to 40,000 spores/mL with distilled deionized water (ddH_2_O) containing 0.004% Tween 20 and sprayed onto leaves (0.01 mL/cm^2^) of ‘Robusta’. After inoculation, the leaves were incubated at 16 °C with 16 h/day illumination. The susceptibility of ‘Robusta’ was tested in laboratory inoculation experiments. The disease score was analyzed based on uredinial pustule area and inoculum density. The samples harvested at different time points with different treatments were immediately snap-frozen in liquid nitrogen for further nucleic acid isolation. According to the procedure described previously [34,35], disease scores were given as scale 0 (slope factor = 0), scale 1 (0 < slope factor ≤ AMSF × 2/7), scale 2 (AMSF × 2/7 < slope factor ≤ AMSF × 4/7), scale 3 (AMSF × 4/7 < slope factor ≤ AMSF × 6/7), and scale 4 (slope factor > AMSF × 6/7).

### 4.2. In Vivo Monitoring of Rust Growth

Total DNA was extracted from 100 mg frozen E4-infected leaf tissues using a DNeasy Plant Mini kit (Qiagen, Valencia, CA, USA) at 0 hours post inoculation (hpi), 2 hpi, 6 hpi, 12 hpi, 1 day post inoculation (dpi), 2 dpi, 3 dpi, 4 dpi, 5 dpi, 6 dpi, and 7 dpi. Ribonuclease A was added to remove RNA during extraction. DNA was quantified by using a NanoDrop (Thermo Scientific, Wilmington, DE, USA). The growth of E4 at each time point was assessed with quantitative PCR (qPCR) via the quantification of its DNA [36,37,38]. A total of 10 ng DNA was used for qPCR amplifications with ITS primers (Table S5) specific for ‘Robusta’ and for E4. Amplifications were performed in the Stratagene Mx3000P qPCR system (Agilent Technologies, Palo Alto, CA, USA). After an initial 5 min activation step at 95 °C, 40 cycles of 94 °C for 15 s, 57 °C for 1 min, and 72 °C for 30 s were performed, and a melting curve was determined at the end of cycling. The pathogen growth was considered as the quantification cycle (Cq) of E4 ITS amplicons quantified in the compatible interaction compared to the Cq of ‘Robusta’ ITS amplicons at different time points [36]. The qPCR results were normalized as 2^−∆Cq^ [31]. Three biological replicates were performed.

### 4.3. Total RNA Preparation

Total RNA was extracted from 100 mg frozen leaf tissues using an optimized cetyltrimethylammonium bromide (CTAB)-based protocol [39]. Total RNA was derived from E4-inoculated leaves (rust+) at 2 hpi, 6 hpi, 12 hpi, 1 dpi, 2 dpi, 4 dpi, and 7 dpi and E4-free leaves (rust-, the control) at the same time points. The time points were chosen according to previous studies [28]. The quality of the extracted RNA was preliminarily assessed by using a BioPhotometer D30 (Eppendorf, Hamburg, Germany). The concentration and integrity of RNA were quantified by using an Agilent 2100 Bioanalyzer (Agilent Technologies). The values of OD_260_ nm/OD_280_ nm and OD_260_ nm/OD_230_ nm were quantified by using a NanoDrop (Thermo Scientific, Wilmington, DE, USA).

### 4.4. Digital Gene Expression (DGE) Library Preparation and Sequencing

DGE libraries representing rust+ and rust- at 12 hpi and 4 dpi were prepared and sequenced according to the Illumina/Solexa standard protocol. Three biological replicates were performed to analyze the differential expression profiles. The DGE libraries were sequenced by using 1 × 36-bp modules and analyzed according to the method of Anders and Huber, 2010 [40]. The annotation and expression of each gene were obtained from the raw data (reads) exported using the Illumina DGE sequencing program (Illumina, San Diego, CA, USA) following data processing (detailed in Text S1).

### 4.5. RT-qPCR of TF Genes

RT-qPCR was performed with the GoTaq 2-Step RT-qPCR System (Promega, Madison, WI, USA) using the Stratagene Mx3000P qPCR system to validate the expression levels of the selected TF genes at 2 hpi, 6 hpi, 12 hpi, 1 dpi, 2 dpi, 4 dpi, and 7 dpi. All primers used in this study, including primers of the control 18S ribosomal RNA [41,42], are listed in Table S5. Two-step RT-qPCR was then performed. The first step was 1 cycle of 95 °C for 2 min. The second step was 40 cycles of 95 °C for 15 s and 60 °C for 1 min. Then, 1 cycle of dissociation was performed from 60 to 95 °C. The normalization of the RT-qPCR results followed the $2^{-\Delta \Delta C_{T}}$ method [43]. Three biological replicates were performed.

### 4.6. Bioinformatics and Statistical Analysis

BLAST homology searches were performed against the *M. larici-populina* genome, and the mapped reads were removed from the rust+ library. The dataset was deposited in the Sequence Read Archive (SRA, accession No. SRR4302070). To compare the differential transcript patterns between the rust- and rust+ libraries of the two time points, we normalized (by the mean of transcripts per million clean reads, TPM) the read distributions for the gene expression levels in each library to construct effective sizes of libraries. To associate the infection of E4 with alterations in poplar gene expression, the differences in gene expression levels were compared based on the log_2_fold changes (rust+/rust-) of the normalized reads. On the basis of the differential expression analysis [40] and the control of the false discovery rate (FDR) [44], differentially expressed genes (DEGs) and unchanged genes were identified.

Interactions and subcellular localizations of proteins were predicted based on the alignment to the subcellular location database for Arabidopsis (SUBA, http://suba.plantenergy.uwa.edu.au) (detailed in Text S2). Gene Ontology (GO) [45,46] analysis and enrichment were performed for the differentially expressed poplar genes with the GO Analysis Toolkit and Database for Agricultural Community (http://bioinfo.cau.edu.cn/agriGO/). Analysis of the overrepresented TF-binding motifs in the promoter sequences was performed as described by Breeze et al., 2011 and Windram et al., 2012. Kyoto Encyclopedia of Genes and Genomes (KEGG) pathways were generated according to KEGG (http://www.genome.jp/kegg/kegg2.html). A. thaliana interologs of differentially expressed poplar genes were obtained from BLAST searches using the criterion of E-value threshold <1e-10. A differentially expressed poplar gene network was predicted based on their interologs with A. thaliana, according to the database of the Bio-Analytic Resource for Plant Biology (http://bar.utoronto.ca/). Visualization of the network was generated using Cytoscape (v.3.7.1, https://cytoscape.org/). A principal component analysis (PCA) assessing the covariance between the 11 time points of rust growth was performed with Paleontological Statistics (Version 3.15, https://folk.uio.no/ohammer/past/).

## 5. Conclusions

The infection with E4 induced a network of TFs where a few TFs responded rapidly and positively. It had a substantial impact on disease-related TF families in the very beginning of the infection and caused further reactions in ‘Robusta’ by upregulating some resistance-related TF genes when E4 was at the biotrophic period. The passive attitude of ‘Robusta’ to E4 infection was mainly manifested as the inhibition of the expression of disease-related TFs and the lack of response of most TFs, especially during the infection phase. The ethylene, jasmonic acid, and auxin pathways were downregulated, while a calcium-binding protein was upregulated. No other significantly changed phytohormone-related genes were found, which was consistent with the pathological process in the absence of an immune response. These results suggested that the lack of response of most TFs during the infection phase of E4 is related to the susceptibility of ‘Robusta’.

## Supplementary Materials

The following are available online at https://www.mdpi.com/1422-0067/20/19/4806/s1, Figure S1: Symptoms of Rust+ ‘Robusta’ leaves at 12 hpi and 4 dpi. (A) 10× close-up of rust+ ‘Robusta’ leaf at 12 hpi. (B) 10× close-up of trypan blue staining on rust+ ‘Robusta’ leaf at 4 dpi.; Figure S2: Differentially expressed genes analysis; Figure S3: GO enrichment for all E4 responsive genes; Figure S4: KEGG pathway and atlas, Table S1: Significantly changed genes following infection with E4.; Table S2: Expression of TFs at 12 hpi and 4 dpi.; Table S3: The network of 5 TF families that regulate potential target genes with their corresponding TF-binding motifs.; Table S4: KEGG reference pathways.; Table S5: Primers used in this study.
