# Supplementary material for: Molecular Characterization of the Transcription Factors in Susceptible Poplar Infected with Virulent Melampsora larici-populina
Source: Int J Mol Sci. 2019 Sep 27;20(19):4806. doi: 10.3390/ijms20194806 (PMC6801979; doi:10.3390/ijms20194806)
Supplement: Supplementary file 1 [file ijms-20-04806-s001.pdf]

Molecular characterization of the transcription factors in susceptible poplar infected with virulent Melampsora larici-populina

Qiaoli Chen [1,§], Jianan Wang [1,§], Danlei Li [1,2,*], Zhiying Wang [1,*], Feng Wang [1,2] and Ruizhi Zhang [1]

1. School of Forestry, Northeast Forestry University, Harbin 150040, Heilongjiang, P. R. China
2. Key Laboratory of Sustainable Forest Ecosystem Management-Ministry of Education, Northeast Forestry University, Harbin 150040, Heilongjiang, P. R. China

[§]These authors contributed equally to this work.

* Corresponding authors: Danlei Li, School of Forestry, Northeast Forestry University, Harbin 150040, China; E-mail: danleili@nefu.edu.cn Zhiying Wang, School of Forestry, Northeast Forestry University, Harbin 150040, China; E-mail: zhiyingwang@nefu.edu.cn

Table S1. Significantly changed genes following infection with E4.

No.	12 hpi Log$_2$(rust/ck)	4 dpi Log$_2$(rust/ck)	Annotation
1	1.83	1.10	2-oxoglutarate (2OG) and Fe(II)-dependent oxygenase superfamily protein
2	-1.21	-1.42	5'-AMP-activated protein kinase-related
3	-1.27	-1.37	ACT domain repeat 4
4	1.25	-1.21	ACT-like superfamily protein
5	-1.15	1.73	adenylate cyclases
6	-1.44	-1.12	aldehyde dehydrogenase 3H1
7	1.43	1.85	Aldolase superfamily protein
8	1.56	-2.07	Aldolase superfamily protein
9	-2.02	2.87	alpha/beta-Hydrolases superfamily protein
10	-1.98	-1.40	alpha/beta-Hydrolases superfamily protein
11	-1.94	4.22	alpha/beta-Hydrolases superfamily protein
12	1.20	1.65	alpha/beta-Hydrolases superfamily protein
13	1.71	1.32	alpha/beta-Hydrolases superfamily protein
14	-1.41	1.10	Ankyrin repeat family protein
15	1.07	-1.37	Ankyrin repeat family protein
16	1.08	-1.71	Ankyrin repeat family protein
17	1.23	1.12	Ankyrin repeat family protein
18	1.08	-2.47	arabinogalactan protein 14
19	1.45	1.27	ARM repeat superfamily protein
20	2.58	1.13	ARM repeat superfamily protein
21	-1.68	-1.44	arogenate dehydrogenase
22	1.06	-1.59	auxin response factor 16
23	1.19	-1.11	Barwin-related endoglucanase
24	-2.04	2.11	basic helix-loop-helix (bHLH) DNA-binding superfamily protein
25	1.03	1.02	basic helix-loop-helix (bHLH) DNA-binding superfamily protein
26	1.13	1.42	beta glucosidase 17
27	-	1.22	beta HLH protein 71
28	-1.08	1.27	Beta-glucosidase, GBA2 type family protein
29	-2.52	-	Bifunctional inhibitor/lipid-transfer protein/seed storage 2S albumin superfamily protein
30	1.09	-4.23	Bifunctional inhibitor/lipid-transfer protein/seed storage 2S albumin superfamily protein
31	-3.13	-	Biotin/lipoate A/B protein ligase family
32	2.40	1.24	BON association protein 2
33	-1.32	1.19	BURP domain-containing protein
34	2.78	1.21	C2H2 and C2HC zinc fingers superfamily protein
35	1.24	-2.06	Calcineurin-like metallo-phosphoesterase superfamily protein
36	1.29	1.19	Calcineurin-like metallo-phosphoesterase superfamily protein
37	1.48	-3.54	Calcineurin-like metallo-phosphoesterase superfamily protein
38	1.12	0.96	Calcium-binding EF-hand family protein
39	1.28	1.19	calmodulin-like 11
40	0.98	2.83	carboxyesterase 18
41	1.05	1.54	cellulose synthase-like B4
42	1.09	-1.64	cellulose synthase-like B4
43	1.09	-1.07	Chalcone and stilbene synthase family protein
44	-1.60	1.49	Chaperone DnaJ-domain superfamily protein
45	-1.47	-1.07	Chaperone DnaJ-domain superfamily protein
46	-1.39	-1.33	Chaperone DnaJ-domain superfamily protein
47	-1.37	1.09	Chaperone DnaJ-domain superfamily protein
48	1.36	1.67	chitinase A
49	1.63	2.11	Chlorophyll A-B binding family protein
50	0.99	1.76	Cobalamin-independent synthase family protein
51	2.19	1.10	Concanavalin A-like lectin protein kinase family protein
52	2.30	2.99	Copper amine oxidase family protein

53	-1.09	-1.29	CP12 domain-containing protein 3
54	2.09	-1.42	cryptdin protein-related
55	0.98	1.21	Cupredoxin superfamily protein
56	1.42	1.41	cysteine synthase D1
57	1.42	2.10	cysteine synthase D1
58	0.98	1.28	cysteine-rich RLK (RECEPTOR-like protein kinase) 25
59	1.21	1.15	cytochrome p450 81d1
60	-	2.47	cytochrome P450, family 707, subfamily A, polypeptide 2
61	1.04	1.06	cytochrome P450, family 71, subfamily B, polypeptide 34
62	1.04	-4.17	cytochrome P450, family 77, subfamily A, polypeptide 4
63	0.99	-1.15	cytochrome P450, family 81, subfamily D, polypeptide 8
64	-1.66	1.02	cytochrome P450, family 82, subfamily G, polypeptide 1
65	-2.25	1.26	cytochrome P450, family 94, subfamily B, polypeptide 3
66	-1.15	-1.72	Deoxyxylulose-5-phosphate synthase
67	1.82	1.02	dicarboxylate carrier 2
68	1.36	1.11	Disease resistance-responsive (dirigent-like protein) family protein
69	2.05	1.67	D-mannose binding lectin protein with Apple-like carbohydrate-binding domain
70	-1.38	2.25	Eukaryotic aspartyl protease family protein
71	-1.63	1.45	Exostosin family protein
72	1.54	1.17	expansin A4
73	1.07	-2.22	extensin 4
74	2.19	1.12	FAD-binding Berberine family protein
75	3.19	-1.48	FAD-binding Berberine family protein
76	2.08	1.04	fatty acid amide hydrolase
77	1.32	1.23	fatty acid desaturase A
78	1.43	-1.60	Fatty acid/sphingolipid desaturase
79	-	-1.84	FKBP-type peptidyl-prolyl cis-trans isomerase family protein
80	2.02	1.31	flavin-dependent monooxygenase 1
81	1.02	-1.09	galactinol synthase 2
82	-1.09	-1.58	galacturonosyltransferase-like 7
83	1.08	1.08	gamma vacuolar processing enzyme
84	1.55	1.41	glutamate receptor 2.7
85	-1.25	1.11	glutathione S-transferase TAU 25
86	-1.24	1.13	glutathione S-transferase TAU 25
87	1.54	1.89	Glycosyl hydrolase family protein with chitinase insertion domain
88	-1.61	1.39	growth-regulating factor 7
89	1.07	1.67	HCO3- transporter family
90	1.40	1.60	HCO3- transporter family
91	1.43	1.45	HCO3- transporter family
92	1.51	1.00	HCO3- transporter family
93	-1.95	-1.86	heat shock transcription factor A2
94	-1.94	-1.26	homeobox protein 2
95	-1.55	1.09	Homeobox-leucine zipper protein 4 (HB-4) / HD-ZIP protein
96	1.03	1.20	Homeodomain-like superfamily protein
97	1.06	1.49	Homeodomain-like superfamily protein
98	1.45	-1.30	Homeodomain-like superfamily protein
99	-	1.43	Homeodomain-like superfamily protein
100	-	1.56	HSP20-like chaperones superfamily protein
101	-1.20	-1.83	HXXXD-type acyl-transferase family protein
102	0.95	1.66	hydroxy methylglutaryl CoA reductase 1
103	1.16	1.77	Integrase-type DNA-binding superfamily protein
104	-3.12	-1.62	jasmonate-zim-domain protein 5
105	-	1.23	jasmonate-zim-domain protein 8
106	2.59	-1.19	kunitz trypsin inhibitor 1
107	2.94	-	kunitz trypsin inhibitor 1
108	-1.26	1.65	laccase 14
109	1.56	-1.54	laccase 17
110	1.21	-1.48	Laccase/Diphenol oxidase family protein

111	1.13	-3.79	Late embryogenesis abundant (LEA) hydroxyproline-rich glycoprotein family
112	-	1.60	Late embryogenesis abundant (LEA) protein
113	1.60	3.06	Late embryogenesis abundant protein (LEA) family protein
114	-1.69	1.07	Leucine-rich repeat (LRR) family protein
115	1.87	1.79	Leucine-rich repeat protein kinase family protein
116	0.98	-1.18	Leucine-rich repeat receptor-like protein kinase family protein
117	1.09	1.19	Leucine-rich repeat receptor-like protein kinase family protein
118	1.57	-1.55	Leucine-rich repeat transmembrane protein kinase protein
119	-1.50	1.46	LOB domain-containing protein 40
120	1.20	1.81	Major facilitator superfamily protein
121	1.36	1.17	Major Facilitator Superfamily with SPX (SYG1/Pho81/XPR1) domain-containing protein
122	1.89	2.77	MATE efflux family protein
123	2.98	-	MATE efflux family protein
124	1.20	1.07	Mitochondrial substrate carrier family protein
125	1.50	2.89	Mitochondrial substrate carrier family protein
126	2.18	-1.43	MLP-like protein 423
127	1.56	6.88	myb domain protein 97
128	1.37	1.04	myo-inositol-1-phosphate synthase 2
129	1.37	-2.03	myo-inositol-1-phosphate synthase 2
130	-1.71	-1.05	Myzus persicae-induced lipase 1
131	-5.44	-1.77	N/A
132	-5.12	-	N/A
133	-5.07	-1.87	N/A
134	-4.85	1.06	N/A
135	-4.27	1.75	hypothetical protein POPTR_0010s18640g
136	-4.03	1.47	hypothetical protein POPTR_0015s08230g
137	-3.94	4.06	N/A
138	-3.94	-	N/A
139	-3.94	-	hypothetical protein POPTR_0005s27400g
140	-3.94	-	N/A
141	-3.70	-1.17	N/A
142	-3.58	1.85	hypothetical protein POPTR_0005s08640g
143	-3.25	1.55	N/A
144	-3.21	1.06	N/A
145	-3.13	1.32	hypothetical protein POPTR_0006s27620g
146	-3.13	1.58	N/A
147	-3.12	1.09	N/A
148	-3.09	1.20	N/A
149	-3.00	-1.43	N/A
150	-3.00	-	hypothetical protein POPTR_0008s05350g
151	-2.95	1.30	N/A
152	-2.93	-	N/A
153	-2.89	-1.51	N/A
154	-2.87	1.21	N/A
155	-2.85	1.22	hypothetical protein POPTR_0005s09440g
156	-2.85	1.79	N/A
157	-2.84	1.50	N/A
158	-2.74	1.08	N/A
159	-2.72	1.71	N/A
160	-2.69	-	PREDICTED: cytochrome P450 94A1-like [*Populus euphratica*]
161	-2.64	0.99	unknown
162	-2.63	1.17	N/A
163	-2.59	-1.64	N/A
164	-2.56	1.45	N/A
165	-2.53	-1.76	N/A
166	-2.46	-1.18	N/A
167	-2.46	-1.25	hypothetical protein POPTR_0015s08290g
168	-2.45	-	N/A

169	-2.44	1.13	hypothetical protein POPTR_0003s06860g
170	-2.41	-1.27	hypothetical protein POPTR_0015s08290g
171	-2.38	1.42	hypothetical protein POPTR_0015s08290g
172	-2.27	1.12	N/A
173	-2.27	1.08	hypothetical protein POPTR_0005s15980g
174	-2.26	-1.21	hypothetical protein POPTR_0015s08290g
175	-2.24	-	N/A
176	-2.24	1.00	N/A
177	-2.23	-1.74	hypothetical protein POPTR_0014s16290g
178	-2.22	3.53	PREDICTED: serine
179	-2.22	1.24	N/A
180	-2.22	1.27	N/A
181	-2.21	-1.24	hypothetical protein POPTR_0001s17670g
182	-2.20	3.54	N/A
183	-2.20	2.47	hypothetical protein POPTR_0004s035802g, partial
184	-2.19	1.37	heat shock protein 17.7
185	-2.18	1.60	N/A
186	-2.17	1.57	MTD1 family protein
187	-2.17	-1.39	hypothetical protein POPTR_0010s21830g
188	-2.16	1.15	hypothetical protein POPTR_0006s10390g
189	-2.16	1.29	N/A
190	-2.14	1.88	hypothetical protein POPTR_0015s08290g
191	-2.13	1.13	hypothetical protein POPTR_0009s04410g
192	-2.13	1.21	N/A
193	-2.12	1.46	N/A
194	-2.12	1.89	N/A
195	-2.10	1.97	hypothetical protein POPTR_0008s19430g
196	-2.07	1.21	hypothetical protein POPTR_0006s06430g
197	-2.07	-1.94	hypothetical protein POPTR_0002s13150g
198	-2.06	-1.24	PREDICTED: serine
199	-2.04	-2.56	N/A
200	-1.97	1.33	N/A
201	-1.97	-4.23	N/A
202	-1.97	1.06	N/A
203	-1.96	1.12	N/A
204	-1.96	3.41	N/A
205	-1.95	-3.04	hypothetical protein POPTR_0014s15800g
206	-1.95	4.90	N/A
207	-1.94	1.25	N/A
208	-1.93	1.57	N/A
209	-1.92	-1.87	homeobox-leucine zipper family protein
210	-1.91	-1.46	hypothetical protein POPTR_0010s16180g
211	-1.91	1.84	homeobox-leucine zipper family protein
212	-1.91	1.38	hypothetical protein POPTR_0014s03820g
213	-1.91	-1.79	N/A
214	-1.91	-1.48	N/A
215	-1.90	1.44	N/A
216	-1.87	-1.53	N/A
217	-1.87	1.04	hypothetical protein POPTR_0001s10160g
218	-1.87	2.37	hypothetical protein POPTR_0010s16180g
219	-1.86	0.99	N/A
220	-1.86	-1.56	N/A
221	-1.85	1.01	N/A
222	-1.84	1.04	hypothetical protein POPTR_0012s10200g
223	-1.84	-1.01	N/A
224	-1.84	1.07	N/A
225	-1.84	1.08	N/A
226	-1.84	2.07	PREDICTED: ankyrin repeat-containing protein At5g02620-like [*Populus euphratica*]
227	-1.84	2.26	N/A

228	-1.83	1.22	hypothetical protein POPTR_0005s09450g
229	-1.83	-1.28	hypothetical protein POPTR_0003s16550g
230	-1.82	1.80	N/A
231	-1.81	1.95	PREDICTED: ankyrin repeat-containing protein At5g02620-like [*Populus euphratica*]
232	-1.81	-2.75	N/A
233	-1.81	1.45	hypothetical protein POPTR_0014s15800g
234	-1.81	1.78	N/A
235	-1.80	-2.13	N/A
236	-1.80	-1.10	N/A
237	-1.80	1.23	hypothetical protein POPTR_0001s15470g
238	-1.79	1.17	N/A
239	-1.79	-5.01	N/A
240	-1.78	1.73	hypothetical protein POPTR_0013s01110g
241	-1.77	1.42	N/A
242	-1.77	-1.36	NADPH-protochlorophyllide oxidoreductase family protein
243	-1.76	-2.35	hypothetical protein POPTR_0008s19430g
244	-1.76	-1.58	N/A
245	-1.76	1.53	N/A
246	-1.76	-1.95	constitutive photomorphogenic 3 family protein
247	-1.75	1.83	N/A
248	-1.74	1.12	hypothetical protein POPTR_0005s09460g
249	-1.73	1.16	invertase
250	-1.72	1.43	PREDICTED: arogenate dehydrogenase 1, chloroplastic-like [*Populus euphratica*]
251	-1.72	1.24	hypothetical protein POPTR_0820s00200g, partial
252	-1.72	1.69	N/A
253	-1.71	-1.55	N/A
254	-1.71	1.71	PREDICTED: calcineurin B-like protein 4 isoform X1 [*Populus euphratica*]
255	-1.70	2.40	N/A
256	-1.67	4.20	zinc finger family protein
257	-1.67	-1.79	hypothetical protein POPTR_0005s147101g, partial
258	-1.67	1.82	hypothetical protein POPTR_0012s14480g
259	-1.67	1.05	unnamed protein product [*Vitis vinifera*]
260	-1.67	1.00	N/A
261	-1.66	1.24	N/A
262	-1.66	1.52	N/A
263	-1.65	1.46	N/A
264	-1.65	-1.61	hypothetical protein POPTR_0017s07770g
265	-1.65	-1.14	hypothetical protein POPTR_0014s15800g
266	-1.64	-2.52	17.4 kDa class III heat shock family protein
267	-1.64	4.28	hypothetical protein POPTR_0006s21150g
268	-1.64	1.14	N/A
269	-1.63	-2.42	hypothetical protein POPTR_0013s03430g
270	-1.63	-1.78	N/A
271	-1.63	-1.34	pentatricopeptide repeat-containing family protein
272	-1.63	1.50	PREDICTED: arogenate dehydrogenase 1, chloroplastic-like [*Populus euphratica*]
273	-1.63	1.06	N/A
274	-1.62	1.60	N/A
275	-1.60	1.03	N/A
276	-1.60	1.18	hypothetical protein POPTR_0011s12270g
277	-1.60	3.05	N/A
278	-1.60	-1.88	PREDICTED: serine
279	-1.59	1.09	N/A
280	-1.59	1.24	hypothetical protein POPTR_0012s01500g
281	-1.59	1.36	hypothetical protein POPTR_0017s07770g
282	-1.58	-2.13	hypothetical protein POPTR_0017s07770g
283	-1.58	1.04	hypothetical protein POPTR_0013s03430g

284	-1.58	2.09	hypothetical protein POPTR_0018s09240g
285	-1.58	1.25	hypothetical protein POPTR_0017s07770g
286	-1.58	-1.21	NADPH-protochlorophyllide oxidoreductase family protein
287	-1.58	1.31	hypothetical protein POPTR_0009s13440g
288	-1.58	1.08	NADPH-protochlorophyllide oxidoreductase family protein
289	-1.58	1.06	N/A
290	-1.57	1.35	NADPH-protochlorophyllide oxidoreductase family protein
291	-1.57	1.56	hypothetical protein POPTR_0001s08310g
292	-1.57	1.23	NADPH-protochlorophyllide oxidoreductase family protein
293	-1.57	1.50	N/A
294	-1.57	1.22	N/A
295	-1.56	1.34	hypothetical protein POPTR_0011s12270g
296	-1.56	1.34	hypothetical protein POPTR_0018s09240g
297	-1.56	1.69	N/A
298	-1.56	1.26	hypothetical protein POPTR_0011s12270g
299	-1.55	1.17	glycosyl hydrolase family 17 family protein
300	-1.55	3.02	ribosomal protein S4 [*Populus alba*]
301	-1.55	1.05	N/A
302	-1.55	-1.25	N/A
303	-1.54	1.51	hypothetical protein POPTR_0010s06320g
304	-1.54	1.25	basic helix-loop-helix family protein
305	-1.53	2.36	N/A
306	-1.53	1.25	hypothetical protein M569_00846 [*Genlisea aurea*]
307	-1.53	-1.27	N/A
308	-1.53	1.05	hypothetical protein POPTR_0010s06320g
309	-1.52	-1.47	hypothetical protein POPTR_0017s07770g
310	-1.52	1.12	PREDICTED: uncharacterized protein LOC105142594 isoform X1 [*Populus euphratica*]
311	-1.51	1.96	N/A
312	-1.50	2.04	N/A
313	-1.50	-3.58	N/A
314	-1.50	-1.39	hypothetical protein POPTR_0001s11500g
315	-1.50	1.89	N/A
316	-1.49	1.06	hypothetical protein POPTR_0820s00200g, partial
317	-1.49	-1.39	hypothetical protein POPTR_0001s08310g
318	-1.49	1.18	pathogenesis-related family protein
319	-1.49	-1.81	N/A
320	-1.48	1.96	N/A
321	-1.47	1.02	PREDICTED: uncharacterized protein LOC105134363 [*Populus euphratica*]
322	-1.47	1.55	N/A
323	-1.47	1.74	hypothetical protein POPTR_0010s09600g
324	-1.46	1.11	hypothetical protein POPTR_0004s20030g
325	-1.46	-1.34	hypothetical protein POPTR_0001s16520g
326	-1.46	1.93	hypothetical protein POPTR_0001s16520g
327	-1.45	1.05	hypothetical protein POPTR_0001s19160g
328	-1.45	-1.34	N/A
329	-1.45	1.21	N/A
330	-1.45	1.10	hypothetical protein POPTR_0001s16520g
331	-1.45	1.64	hypothetical protein POPTR_0008s05890g
332	-1.45	-1.65	N/A
333	-1.45	1.90	PREDICTED: uncharacterized protein LOC105142594 isoform X1 [*Populus euphratica*]
334	-1.44	-1.62	N/A
335	-1.44	1.11	hypothetical protein POPTR_0017s02810g
336	-1.43	1.07	N/A
337	-1.43	-2.16	hypothetical protein POPTR_0001s16520g
338	-1.42	-1.57	hypothetical protein POPTR_0001s16520g
339	-1.42	-1.09	hypothetical protein POPTR_0007s09900g
340	-1.42	-2.08	hypothetical protein POPTR_0014s11700g

341	-1.41	-1.49	hypothetical protein POPTR_0008s05890g
342	-1.41	1.99	N/A
343	-1.41	-1.46	hypothetical protein POPTR_0017s02810g
344	-1.40	-1.18	hypothetical protein POPTR_0003s21260g
345	-1.40	1.27	hypothetical protein POPTR_0008s19020g
346	-1.40	-1.75	hypothetical protein POPTR_0001s19160g
347	-1.40	-1.14	N/A
348	-1.40	2.11	hypothetical protein POPTR_0001s19160g
349	-1.39	1.11	hypothetical protein POPTR_0014s11700g
350	-1.39	-1.36	N/A
351	-1.39	1.03	hypothetical protein POPTR_0008s19020g
352	-1.39	1.28	N/A
353	-1.39	1.50	hypothetical protein POPTR_0008s19020g
354	-1.38	1.52	leucine-rich repeat family protein
355	-1.38	1.37	glycosyl hydrolase family 17 family protein
356	-1.37	-1.75	N/A
357	-1.37	1.41	N/A
358	-1.36	1.08	hypothetical protein POPTR_0002s04900g
359	-1.35	-1.99	mangrin family protein
360	-1.35	1.38	hypothetical protein POPTR_0014s11700g
361	-1.35	-1.17	N/A
362	-1.35	1.21	hypothetical protein POPTR_0012s11110g
363	-1.34	1.80	N/A
364	-1.34	1.45	N/A
365	-1.34	-1.47	hypothetical protein POPTR_0002s04900g
366	-1.34	-1.40	1-phosphatidylinositol phosphodiesterase-related family protein
367	-1.34	-1.31	N/A
368	-1.34	-1.09	N/A
369	-1.33	1.29	hypothetical protein POPTR_0008s19020g
370	-1.33	1.70	N/A
371	-1.33	1.37	N/A
372	-1.33	1.33	N/A
373	-1.33	-1.80	N/A
374	-1.32	1.27	hypothetical protein POPTR_0004s14410g
375	-1.32	2.69	hypothetical protein POPTR_0008s19020g
376	-1.32	1.75	hypothetical protein POPTR_0008s19020g
377	-1.32	1.37	brassinosteroid-responsive ring-H2 family protein
378	-1.32	2.65	N/A
379	-1.32	-1.57	N/A
380	-1.32	2.22	N/A
381	-1.31	1.18	hypothetical protein POPTR_0008s19020g
382	-1.31	-1.72	N/A
383	-1.31	-1.15	N/A
384	-1.31	0.97	N/A
385	-1.31	-1.35	N/A
386	-1.31	-1.30	N/A
387	-1.31	1.01	hypothetical protein POPTR_0014s11700g
388	-1.30	1.60	N/A
389	-1.30	-1.33	N/A
390	-1.30	1.72	N/A
391	-1.29	1.01	N/A
392	-1.29	0.97	glycosyl hydrolase family 17 family protein
393	-1.29	1.34	hypothetical protein POPTR_0008s19020g
394	-1.29	1.82	hypothetical protein POPTR_0002s02220g
395	-1.29	-1.56	mangrin family protein
396	-1.28	-1.29	hypothetical protein POPTR_0016s14790g
397	-1.28	-1.79	N/A
398	-1.28	-1.16	N/A
399	-1.28	1.86	hypothetical protein POPTR_0004s17810g
400	-1.27	3.43	N/A

401	-1.27	-1.75	N/A
402	-1.27	1.23	N/A
403	-1.27	1.58	N/A
404	-1.27	1.80	N/A
405	-1.26	1.14	17.5 kd heat shock family protein
406	-1.26	-1.44	N/A
407	-1.26	-2.19	hypothetical protein POPTR_0001s19160g
408	-1.26	-1.85	N/A
409	-1.25	1.15	hypothetical protein POPTR_0004s14430g
410	-1.25	1.28	aspartyl protease family protein
411	-1.25	1.22	N/A
412	-1.25	1.26	N/A
413	-1.25	1.36	N/A
414	-1.25	1.16	hypothetical protein POPTR_0001s39680g
415	-1.25	1.08	N/A
416	-1.24	-1.24	hypothetical protein POPTR_0010s04360g
417	-1.24	-1.20	hypothetical protein POPTR_0001s39920g
418	-1.24	1.04	hypothetical protein POPTR_2015s00200g
419	-1.24	-1.30	unknown [*Populus trichocarpa* x *Populus deltoides*]
420	-1.24	1.10	N/A
421	-1.23	1.25	PREDICTED: transcription factor LHW-like isoform X2 [*Populus euphratica*]
422	-1.23	1.84	PREDICTED: uncharacterized protein LOC105132412 isoform X1 [*Populus euphratica*]
423	-1.23	1.88	N/A
424	-1.23	-2.22	N/A
425	-1.23	1.85	N/A
426	-1.22	0.96	hypothetical protein POPTR_0001s39680g
427	-1.22	1.09	N/A
428	-1.22	1.31	hypothetical protein POPTR_0001s39680g
429	-1.22	-1.35	hypothetical protein POPTR_0004s14400g
430	-1.21	1.50	hypothetical protein POPTR_0013s11000g
431	-1.21	1.79	N/A
432	-1.21	-3.20	hypothetical protein POPTR_0013s11000g
433	-1.20	-2.03	N/A
434	-1.20	-1.57	N/A
435	-1.20	-3.04	hypothetical protein POPTR_0013s11000g
436	-1.19	1.51	hypothetical protein POPTR_0003s13080g
437	-1.19	-1.96	PREDICTED: uncharacterized protein LOC105132412 isoform X1 [*Populus euphratica*]
438	-1.18	1.55	hypothetical protein POPTR_0001s39660g
439	-1.18	1.70	PIGMENT DEFECTIVE EMBRYO family protein
440	-1.18	1.17	mangrin family protein
441	-1.17	1.09	hypothetical protein POPTR_0013s07220g
442	-1.17	1.62	N/A
443	-1.17	1.62	PREDICTED: putative formamidase C869.04 isoform X4 [*Nelumbo nucifera*]
444	-1.17	1.85	methyladenine glycosylase family protein
445	-1.17	1.13	hypothetical protein POPTR_0016s05660g
446	-1.17	1.20	hypothetical protein POPTR_0001s39940g
447	-1.17	2.11	N/A
448	-1.17	1.40	N/A
449	-1.17	1.82	hypothetical protein POPTR_0010s24410g
450	-1.17	1.48	N/A
451	-1.16	-1.39	putative histidine-containing phosphotransfer protein 2
452	-1.16	2.01	hypothetical protein POPTR_0015s00670g
453	-1.16	1.12	N/A
454	-1.15	1.98	N/A
455	-1.15	1.35	hypothetical protein POPTR_0019s13960g, partial
456	-1.14	1.26	N/A

457	-1.14	-1.79	N/A
458	-1.13	-1.11	hypothetical protein POPTR_0002s05050g
459	-1.13	-1.11	N/A
460	-1.13	-1.27	hypothetical protein POPTR_0019s10160g
461	-1.13	1.42	N/A
462	-1.12	2.16	hypothetical protein POPTR_0019s10160g
463	-1.11	-1.29	hypothetical protein POPTR_0016s13840g
464	-1.11	-2.80	hypothetical protein POPTR_0015s00670g
465	-1.11	-1.73	N/A
466	-1.10	1.15	N/A
467	-1.10	4.47	hypothetical protein POPTR_0019s13900g
468	-1.09	3.47	AP2 domain-containing transcription factor family protein
469	-1.09	-2.23	N/A
470	-1.08	1.27	AP2 domain-containing transcription factor family protein
471	-1.08	1.45	N/A
472	-1.08	1.13	hypothetical protein POPTR_0019s10160g
473	0.95	1.05	hypothetical protein POPTR_0011s07850g
474	0.95	-1.61	N/A
475	0.95	2.10	N/A
476	0.95	3.31	N/A
477	0.95	1.21	hypothetical protein POPTR_0012s03210g
478	0.95	1.12	hypothetical protein POPTR_0010s24810g
479	0.96	1.39	hypothetical protein POPTR_0003s10660g
480	0.96	-1.66	hypothetical protein POPTR_0009s11990g
481	0.96	1.00	PREDICTED: uncharacterized protein LOC105793039 [*Gossypium raimondii*]
482	0.96	1.64	N/A
483	0.96	1.80	N/A
484	0.96	2.96	aspartyl protease family protein
485	0.96	-2.25	N/A
486	0.96	1.20	hypothetical protein POPTR_0019s02210g
487	0.96	-1.24	N/A
488	0.96	-1.30	hypothetical protein POPTR_0007s15210g
489	0.96	-1.89	hypothetical protein POPTR_0015s06230g
490	0.96	1.45	N/A
491	0.97	1.39	N/A
492	0.97	-1.75	omega-3 desaturase family protein
493	0.97	1.03	Chain A family protein
494	0.97	1.63	N/A
495	0.97	1.77	N/A
496	0.97	3.10	N/A
497	0.97	1.04	hypothetical protein POPTR_0019s02210g
498	0.97	1.41	N/A
499	0.97	0.99	hypothetical protein POPTR_0004s02820g
500	0.97	1.01	N/A
501	0.97	-3.57	N/A
502	0.97	1.27	harpin-induced family protein
503	0.97	-1.25	N/A
504	0.98	2.47	hypothetical protein POPTR_0015s06230g
505	0.98	1.66	hypothetical protein POPTR_0007s15210g
506	0.98	1.21	hypothetical protein POPTR_0008s15170g
507	0.98	-1.13	cytochrome P450 family protein
508	0.98	-1.79	pathogenesis related protein-1 [*Populus tomentosa*]
509	0.98	2.17	N/A
510	0.98	-1.58	N/A
511	0.98	-1.90	PREDICTED: WD repeat-containing protein tag-125-like [*Populus euphratica*]
512	0.98	1.23	high affinity sulfate transporter family protein
513	0.98	-1.14	hypothetical protein POPTR_0019s13790g
514	0.98	3.84	hypothetical protein POPTR_0008s20300g

515	0.98	1.62	N/A
516	0.98	2.20	hypothetical protein POPTR_0012s09650g
517	0.99	1.56	hypothetical protein POPTR_0015s06230g
518	0.99	1.40	hypothetical protein POPTR_0009s15610g
519	0.99	1.36	PREDICTED: 5-methyltetrahydropteroyltriglutamate--homocysteine methyltransferase 1 isoform X1 [*Populus euphratica*]
520	0.99	-1.21	hypothetical protein POPTR_0006s05480g
521	0.99	3.14	N/A
522	0.99	-3.96	PREDICTED: 5-methyltetrahydropteroyltriglutamate--homocysteine methyltransferase 1 isoform X1 [*Populus euphratica*]
523	0.99	-1.24	hypothetical protein POPTR_0013s03490g
524	0.99	1.54	hypothetical protein POPTR_0006s06740g
525	0.99	4.37	hypothetical protein POPTR_0010s16120g
526	0.99	1.47	N/A
527	0.99	2.37	PREDICTED: 5-methyltetrahydropteroyltriglutamate--homocysteine methyltransferase 1 isoform X1 [*Populus euphratica*]
528	0.99	-2.49	PREDICTED: receptor-like protein 12 [*Populus euphratica*]
529	0.99	-1.77	PREDICTED: 5-methyltetrahydropteroyltriglutamate--homocysteine methyltransferase 1 isoform X1 [*Populus euphratica*]
530	0.99	-1.16	hypothetical protein POPTR_0011s05630g
531	0.99	2.85	plasma membrane intrinsic protein 1-1
532	0.99	1.09	N/A
533	0.99	1.39	PREDICTED: 5-methyltetrahydropteroyltriglutamate--homocysteine methyltransferase 1 isoform X1 [*Populus euphratica*]
534	0.99	1.10	hypothetical protein POPTR_0019s14180g
535	1.00	1.45	PREDICTED: 5-methyltetrahydropteroyltriglutamate--homocysteine methyltransferase 1 isoform X1 [*Populus euphratica*]
536	1.00	1.27	PREDICTED: 5-methyltetrahydropteroyltriglutamate--homocysteine methyltransferase 1 isoform X1 [*Populus euphratica*]
537	1.00	3.03	hypothetical protein POPTR_0005s24970g
538	1.00	1.10	N/A
539	1.00	1.43	N/A
540	1.00	-1.53	hypothetical protein POPTR_0019s14180g
541	1.00	-3.61	N/A
542	1.00	-1.98	N/A
543	1.00	-1.27	hypothetical protein POPTR_0008s15170g
544	1.00	-1.19	N/A
545	1.00	1.15	hypothetical protein POPTR_0003s17220g
546	1.00	1.52	N/A
547	1.00	2.84	PREDICTED: 5-methyltetrahydropteroyltriglutamate--homocysteine methyltransferase 1 isoform X1 [*Populus euphratica*]
548	1.00	-1.30	PREDICTED: 5-methyltetrahydropteroyltriglutamate--homocysteine methyltransferase 1 isoform X1 [*Populus euphratica*]
549	1.00	2.00	N/A
550	1.00	1.20	hypothetical protein POPTR_0005s24970g
551	1.00	2.43	hypothetical protein POPTR_0016s12480g
552	1.00	-2.92	N/A
553	1.01	-1.85	PREDICTED: 5-methyltetrahydropteroyltriglutamate--homocysteine methyltransferase 1 isoform X1 [*Populus euphratica*]
554	1.01	-1.62	hypothetical protein POPTR_0013s05850g

555	1.01	1.92	Pto-like kinase OGw family protein
556	1.01	-1.27	N/A
557	1.01	1.28	N/A
558	1.01	3.26	N/A
559	1.01	-1.68	hypothetical protein POPTR_0005s18480g
560	1.01	1.59	hypothetical protein POPTR_0005s24970g
561	1.02	3.55	PREDICTED: 5-methyltetrahydropteroyltriglutamate--homocysteine methyltransferase 1 isoform X1 [*Populus euphratica*]
562	1.02	-1.91	hypothetical protein POPTR_0014s17100g
563	1.02	1.47	hypothetical protein POPTR_0011s12100g
564	1.02	1.70	PREDICTED: 5-methyltetrahydropteroyltriglutamate--homocysteine methyltransferase 1 isoform X1 [*Populus euphratica*]
565	1.02	-1.24	PREDICTED: F-box
566	1.02	-1.15	hypothetical protein POPTR_0001s35030g
567	1.03	1.07	hypothetical protein POPTR_0030s00520g
568	1.03	-1.94	unknown
569	1.03	1.08	hypothetical protein POPTR_0019s11250g, partial
570	1.03	-1.26	hypothetical protein POPTR_0012s09650g
571	1.03	1.14	N/A
572	1.03	1.54	EXS family protein
573	1.04	-1.51	glycosyl transferase family 17 family protein
574	1.04	1.69	N/A
575	1.04	-1.57	WRKY family transcription factor family protein
576	1.04	1.39	hypothetical protein POPTR_0013s05850g
577	1.04	1.27	N/A
578	1.04	1.45	hypothetical protein POPTR_0013s03060g
579	1.04	-1.44	S-adenosylmethionine synthetase family protein
580	1.04	1.53	asparaginyl endopeptidase family protein
581	1.04	-2.44	naregenin-chalcone synthase family protein
582	1.04	1.93	PREDICTED: glutamate receptor 2.9-like isoform X1 [*Populus euphratica*]
583	1.04	1.18	hypothetical protein POPTR_0004s05880g
584	1.04	1.25	S-adenosylmethionine synthetase family protein
585	1.04	1.57	EXS family protein
586	1.05	1.11	hypothetical protein POPTR_0013s05850g
587	1.05	1.69	hypothetical protein POPTR_0010s24810g
588	1.05	1.10	hypothetical protein POPTR_0013s05850g
589	1.05	-1.10	hypothetical protein POPTR_0013s05850g
590	1.05	1.34	hypothetical protein POPTR_0013s05850g
591	1.05	1.41	hypothetical protein POPTR_0009s15650g
592	1.05	-1.21	hypothetical protein POPTR_0009s11990g
593	1.06	1.35	hypothetical protein POPTR_0006s15580g
594	1.06	1.97	hypothetical protein POPTR_0014s17100g
595	1.06	1.30	hypothetical protein POPTR_0007s15210g
596	1.06	1.51	N/A
597	1.06	1.79	hypothetical protein POPTR_0013s05850g
598	1.06	1.00	hypothetical protein POPTR_0001s22920g
599	1.06	1.38	N/A
600	1.06	1.18	N/A
601	1.07	1.10	N/A
602	1.07	1.12	N/A
603	1.07	-1.52	hypothetical protein POPTR_0009s16430g
604	1.07	-1.20	N/A
605	1.07	1.91	hypothetical protein POPTR_0018s08180g
606	1.08	-1.23	hypothetical protein POPTR_0014s17100g
607	1.08	1.26	hypothetical protein POPTR_0008s20300g
608	1.08	1.25	PREDICTED: glycine-rich cell wall structural protein 1-like [*Populus euphratica*]

609	1.08	1.04	N/A
610	1.08	-1.57	N/A
611	1.08	-1.64	N/A
612	1.08	1.07	hypothetical protein POPTR_0018s05260g
613	1.08	1.03	N/A
614	1.08	0.98	hypothetical protein POPTR_0019s12390g
615	1.08	1.10	hypothetical protein POPTR_0012s00800g
616	1.09	-2.18	N/A
617	1.09	-3.89	N/A
618	1.09	1.64	hypothetical protein POPTR_0018s04650g
619	1.09	-1.50	fasciclin-like AGP 13 family protein
620	1.09	1.21	N/A
621	1.09	-1.35	N/A
622	1.09	1.13	asparaginyl endopeptidase family protein
623	1.09	2.28	hypothetical protein POPTR_0001s14420g
624	1.10	1.30	N/A
625	1.10	-1.22	N/A
626	1.10	-2.38	dihydroflavonol reductase family protein
627	1.10	-2.63	hypothetical protein POPTR_0008s19950g
628	1.11	1.28	asparaginyl endopeptidase family protein
629	1.11	1.67	asparaginyl endopeptidase family protein
630	1.11	1.03	N/A
631	1.11	1.90	asparaginyl endopeptidase family protein
632	1.11	1.18	peroxidase family protein
633	1.11	1.74	hypothetical protein POPTR_0006s06650g
634	1.11	-1.51	hypothetical protein POPTR_0006s06650g
635	1.11	1.00	asparaginyl endopeptidase family protein
636	1.11	-1.68	asparaginyl endopeptidase family protein
637	1.11	-1.26	asparaginyl endopeptidase family protein
638	1.11	1.79	putative sorbitol transporter family protein
639	1.11	1.32	N/A
640	1.12	-3.16	N/A
641	1.12	-1.09	N/A
642	1.12	0.98	hypothetical protein POPTR_0011s12090g
643	1.12	-1.13	N/A
644	1.13	1.01	hypothetical protein POPTR_0015s00580g
645	1.13	1.42	N/A
646	1.13	1.24	dihydroflavonol reductase family protein
647	1.13	-2.41	N/A
648	1.13	1.94	N/A
649	1.13	-1.32	PREDICTED: LOW QUALITY PROTEIN: probable WRKY transcription factor 33 [*Populus euphratica*]
650	1.13	-1.21	PREDICTED: RING-H2 finger protein ATL46-like [*Populus euphratica*]
651	1.13	-1.39	hypothetical protein POPTR_0003s02940g
652	1.14	1.21	hypothetical protein POPTR_0003s06510g
653	1.14	-1.28	N/A
654	1.14	1.16	hypothetical protein POPTR_0008s13300g
655	1.14	1.07	N/A
656	1.14	1.41	N/A
657	1.14	1.24	N/A
658	1.14	1.18	N/A
659	1.14	-2.31	N/A
660	1.15	1.97	hypothetical protein POPTR_0008s06900g
661	1.15	1.21	hypothetical protein POPTR_0001s22900g
662	1.15	1.42	hypothetical protein POPTR_0013s06290g
663	1.15	1.40	hypothetical protein POPTR_0008s13300g
664	1.15	1.17	N/A
665	1.15	-1.64	Histone core [*Cynara cardunculus* var. scolymus]
666	1.15	1.57	N/A

667	1.15	1.55	hypothetical protein POPTR_0004s14710g
668	1.15	1.32	N/A
669	1.15	1.06	N/A
670	1.15	-1.57	N/A
671	1.15	1.22	hypothetical protein POPTR_0019s12380g
672	1.15	1.70	N/A
673	1.15	1.17	hypothetical protein CICLE_v10031656mg [*Citrus clementina*]
674	1.15	2.01	N/A
675	1.15	1.58	auxin-responsive family protein
676	1.16	-1.86	N/A
677	1.16	1.35	hypothetical protein POPTR_0004s14710g
678	1.16	3.03	hypothetical protein POPTR_0004s14710g
679	1.16	-1.62	N/A
680	1.16	1.72	N/A
681	1.16	1.06	hypothetical protein POPTR_0003s06510g
682	1.16	1.15	N/A
683	1.17	1.17	hypothetical protein POPTR_0015s04330g, partial
684	1.17	1.92	N/A
685	1.17	1.20	N/A
686	1.17	1.31	hypothetical protein POPTR_0015s11510g
687	1.17	2.25	hypothetical protein POPTR_0002s19450g
688	1.17	1.68	N/A
689	1.18	1.33	N/A
690	1.18	-1.58	hypothetical protein POPTR_0009s14170g
691	1.18	1.16	hypothetical protein POPTR_0018s08180g
692	1.18	1.69	hypothetical protein POPTR_0004s02040g
693	1.18	1.34	6a-hydroxymaackiain methyltransferase family protein
694	1.18	1.34	CCR4-NOT transcription complex family protein
695	1.19	1.05	PREDICTED: bidirectional sugar transporter SWEET17 isoform X1 [*Populus euphratica*]
696	1.19	1.72	hypothetical protein POPTR_0005s27850g
697	1.19	3.50	N/A
698	1.19	1.12	N/A
699	1.19	-2.49	N/A
700	1.19	1.02	lectin protein kinase
701	1.19	1.50	hypothetical protein POPTR_0005s22630g
702	1.19	1.20	N/A
703	1.20	1.11	hypothetical protein POPTR_0001s45400g
704	1.20	-2.09	PREDICTED: protein trichome birefringence-like 33 [*Populus euphratica*]
705	1.20	-3.02	N/A
706	1.20	-1.27	plastocyanin-like domain-containing family protein
707	1.20	1.02	N/A
708	1.20	1.59	PREDICTED: protein trichome birefringence-like 33 [*Populus euphratica*]
709	1.20	1.56	hypothetical protein POPTR_0002s22280g
710	1.20	1.60	N/A
711	1.20	1.24	N/A
712	1.20	1.91	anion exchange family protein
713	1.20	-1.07	N/A
714	1.20	-1.96	N/A
715	1.21	-1.44	hypothetical protein POPTR_0001s45400g
716	1.21	1.15	PREDICTED: RING-H2 finger protein ATL46-like [*Populus euphratica*]
717	1.21	-1.57	hypothetical protein POPTR_0007s06890g
718	1.21	1.31	hypothetical protein POPTR_0014s04130g
719	1.21	1.69	N/A
720	1.21	-	hypothetical protein POPTR_0012s12090g
721	1.22	1.03	hypothetical protein POPTR_0003s06510g
722	1.22	1.40	hypothetical protein POPTR_0019s07690g

723	1.22	-1.59	hypothetical protein POPTR_0001s22870g
724	1.22	1.12	N/A
725	1.22	1.46	hypothetical protein POPTR_0009s07700g
726	1.23	-1.76	hypothetical protein POPTR_0001s42270g
727	1.23	1.47	N/A
728	1.23	1.17	N/A
729	1.23	1.79	N/A
730	1.24	1.36	N/A
731	1.24	1.45	PREDICTED: protein ROOT HAIR DEFECTIVE 3 homolog 2-like [*Populus euphratica*]
732	1.24	1.61	N/A
733	1.24	-1.08	N/A
734	1.24	1.13	hypothetical protein POPTR_0008s13300g
735	1.24	1.67	hypothetical protein POPTR_0001s22850g
736	1.24	-3.69	sucrose-phosphate synthase family protein
737	1.24	1.66	hypothetical protein POPTR_0016s05260g
738	1.25	1.16	N/A
739	1.25	1.51	N/A
740	1.25	1.60	hypothetical protein POPTR_0001s44380g
741	1.25	-2.12	chloroplast omega-3 desaturase family protein
742	1.25	1.75	hypothetical protein POPTR_0003s05980g
743	1.25	1.24	PREDICTED: glutamate receptor 2.9-like isoform X1 [*Populus euphratica*]
744	1.26	2.86	hypothetical protein POPTR_0013s01190g
745	1.26	2.08	N/A
746	1.26	1.59	hypothetical protein CICLE_v10031656mg [*Citrus clementina*]
747	1.26	1.14	N/A
748	1.27	-1.49	N/A
749	1.27	-1.73	hypothetical protein POPTR_0004s14710g
750	1.27	1.11	hypothetical protein POPTR_0001s45390g
751	1.27	-1.07	Mitochondrial carnitine
752	1.27	2.02	hypothetical protein POPTR_0004s10520g
753	1.27	1.67	N/A
754	1.27	1.19	N/A
755	1.27	1.72	transferase family protein
756	1.27	1.40	Mitochondrial carnitine
757	1.28	-2.12	N/A
758	1.28	-1.09	sucrose-phosphate synthase family protein
759	1.28	1.17	N/A
760	1.28	1.34	N/A
761	1.29	1.06	N/A
762	1.29	-2.13	N/A
763	1.29	0.96	hypothetical protein POPTR_0004s14710g
764	1.29	-1.14	hypothetical protein POPTR_0001s22850g
765	1.29	-1.89	N/A
766	1.29	1.27	PREDICTED: LOW QUALITY PROTEIN: receptor-like protein kinase FERONIA [*Populus euphratica*]
767	1.29	2.88	hypothetical protein POPTR_0016s08050g
768	1.29	2.02	PREDICTED: aldose 1-epimerase-like [*Populus euphratica*]
769	1.30	-1.00	hypothetical protein POPTR_0004s14710g
770	1.30	1.01	Mitochondrial carnitine
771	1.30	1.33	PREDICTED: glutamate receptor 2.9-like isoform X1 [*Populus euphratica*]
772	1.30	1.08	hypothetical protein POPTR_0008s12410g, partial
773	1.30	1.14	N/A
774	1.31	-0.98	N/A
775	1.31	1.35	chloroplast omega-3 desaturase family protein
776	1.31	1.10	N/A
777	1.31	-4.82	hypothetical protein POPTR_0005s13020g
778	1.31	1.34	hypothetical protein POPTR_0005s13020g

779	1.32	-1.20	N/A
780	1.32	-1.82	hypothetical protein POPTR_0001s22850g
781	1.32	-1.06	N/A
782	1.32	-1.45	hypothetical protein POPTR_0009s01490g
783	1.32	-2.19	N/A
784	1.32	1.10	hypothetical protein POPTR_0004s23330g
785	1.33	1.82	N/A
786	1.33	-1.19	hypothetical protein POPTR_0002s12610g
787	1.33	-1.10	calcineurin-like phosphoesterase family protein
788	1.34	1.47	hypothetical protein POPTR_0001s22930g
789	1.34	-2.80	hypothetical protein POPTR_0005s27850g
790	1.34	1.44	N/A
791	1.34	1.68	N/A
792	1.34	1.05	hypothetical protein POPTR_0013s00850g
793	1.35	-1.07	N/A
794	1.35	1.65	N/A
795	1.36	-	N/A
796	1.36	1.48	exostosin family protein
797	1.36	-1.40	N/A
798	1.36	1.81	hypothetical protein POPTR_0004s15020g
799	1.36	2.69	N/A
800	1.36	2.30	hypothetical protein POPTR_0001s22960g
801	1.36	2.00	N/A
802	1.36	1.40	N/A
803	1.37	-	N/A
804	1.37	1.80	N/A
805	1.37	1.13	hypothetical protein POPTR_0001s41170g
806	1.37	1.27	N/A
807	1.37	1.33	N/A
808	1.37	-1.31	hypothetical protein POPTR_0001s41740g
809	1.37	-1.23	N/A
810	1.37	1.46	N/A
811	1.37	1.04	N/A
812	1.37	-1.15	N/A
813	1.37	1.58	N/A
814	1.38	1.42	N/A
815	1.38	-1.04	N/A
816	1.38	1.19	N/A
817	1.38	3.37	N/A
818	1.38	1.54	N/A
819	1.38	-1.41	N/A
820	1.38	1.54	hypothetical protein POPTR_0006s06650g
821	1.38	2.06	hypothetical protein POPTR_0001s45940g
822	1.38	1.13	N/A
823	1.38	0.98	exostosin family protein
824	1.39	1.10	N/A
825	1.39	-1.13	N/A
826	1.39	1.47	N/A
827	1.39	-1.96	N/A
828	1.39	-1.36	N/A
829	1.39	-1.66	N/A
830	1.39	-1.42	chloroplast omega-3 desaturase family protein
831	1.39	1.53	N/A
832	1.39	1.48	N/A
833	1.39	1.88	N/A
834	1.39	-1.49	invertase
835	1.40	1.25	N/A
836	1.40	-1.63	N/A
837	1.40	1.42	hypothetical protein POPTR_0015s03820g
838	1.40	1.48	N/A

839	1.40	-2.13	naregenin-chalcone synthase family protein
840	1.41	2.34	exostosin family protein
841	1.41	-2.28	Histone core [*Cynara cardunculus* var. scolymus]
842	1.41	-1.05	hypothetical protein POPTR_0011s15350g
843	1.41	1.15	hypothetical protein POPTR_0012s08340g
844	1.42	-1.52	N/A
845	1.42	-1.57	N/A
846	1.42	1.52	N/A
847	1.42	-2.17	N/A
848	1.42	-1.36	N/A
849	1.42	1.37	hypothetical protein POPTR_0017s01380g
850	1.43	-1.53	N/A
851	1.43	-1.38	GDSL-motif lipase
852	1.43	1.52	hypothetical protein POPTR_0011s16190g
853	1.43	1.35	N/A
854	1.43	-1.50	hypothetical protein POPTR_0014s06050g
855	1.43	-1.07	N/A
856	1.43	1.12	hypothetical protein POPTR_0018s05760g
857	1.44	-1.25	hypothetical protein POPTR_0243s00200g
858	1.44	1.31	N/A
859	1.44	1.34	hypothetical protein POPTR_0012s08340g
860	1.44	-1.04	hypothetical protein POPTR_0012s08340g
861	1.45	1.24	no apical meristem family protein
862	1.46	-1.38	N/A
863	1.46	-1.83	calcineurin-like phosphoesterase family protein
864	1.46	-1.54	N/A
865	1.46	-1.09	N/A
866	1.46	1.60	N/A
867	1.46	-2.82	N/A
868	1.47	-1.21	hypothetical protein POPTR_0012s08340g
869	1.47	1.67	fatty acid desaturase
870	1.47	-1.08	hypothetical protein POPTR_0015s03820g
871	1.47	1.01	PREDICTED: flavanone 3-dioxygenase-like [*Populus euphratica*]
872	1.47	-2.81	hypothetical protein POPTR_0010s08610g
873	1.48	1.55	N/A
874	1.48	4.56	hypothetical protein POPTR_0018s12710g
875	1.48	1.59	N/A
876	1.48	2.34	N/A
877	1.48	1.11	hypothetical protein POPTR_0006s24480g
878	1.48	1.69	N/A
879	1.48	4.03	N/A
880	1.49	1.10	hypothetical protein POPTR_0017s01380g
881	1.49	-1.19	N/A
882	1.49	-1.41	N/A
883	1.50	-1.77	PREDICTED: flavanone 3-dioxygenase-like [*Populus euphratica*]
884	1.50	-1.13	N/A
885	1.51	-1.83	N/A
886	1.51	1.33	PREDICTED: probable carboxylesterase 15 [*Populus euphratica*]
887	1.52	1.77	N/A
888	1.52	1.07	unknown
889	1.52	-1.38	N/A
890	1.52	1.52	N/A
891	1.52	1.34	hypothetical protein POPTR_0016s12400g
892	1.53	-1.10	N/A
893	1.53	1.05	N/A
894	1.53	1.11	N/A
895	1.53	-4.67	N/A
896	1.54	1.07	hypothetical protein POPTR_0007s09650g
897	1.54	-1.35	Mitochondrial carnitine
898	1.54	1.74	hypothetical protein POPTR_0003s05970g

899	1.54	1.04	hypothetical protein POPTR_0004s21730g
900	1.54	-1.55	N/A
901	1.55	1.09	naregenin-chalcone synthase family protein
902	1.55	1.12	hypothetical protein POPTR_0009s04720g
903	1.55	1.34	hypothetical protein POPTR_0003s21140g
904	1.55	1.13	PREDICTED: nudix hydrolase 8-like isoform X3 [*Populus euphratica*]
905	1.56	1.06	N/A
906	1.56	2.12	N/A
907	1.56	-1.03	Mitochondrial carnitine
908	1.56	-1.31	N/A
909	1.56	1.18	N/A
910	1.57	1.21	unknown
911	1.57	1.38	N/A
912	1.57	2.13	N/A
913	1.57	1.57	PREDICTED: G-type lectin S-receptor-like serine
914	1.58	1.46	PREDICTED: uncharacterized protein LOC105135011 [*Populus euphratica*]
915	1.58	1.43	hypothetical protein POPTR_0018s00270g
916	1.58	-1.81	hypothetical protein POPTR_0004s05180g
917	1.58	-1.43	N/A
918	1.59	-5.39	hypothetical protein POPTR_0008s20740g
919	1.60	-1.35	hypothetical protein POPTR_0013s12010g
920	1.60	-1.24	hypothetical protein POPTR_0002s07630g
921	1.60	1.57	hypothetical protein POPTR_0018s03050g
922	1.60	1.14	hypothetical protein POPTR_0017s07250g
923	1.60	0.98	hypothetical protein POPTR_0007s09650g
924	1.61	-1.66	N/A
925	1.61	3.17	hypothetical protein POPTR_0014s02900g
926	1.62	-1.74	N/A
927	1.62	1.44	N/A
928	1.62	-1.89	N/A
929	1.62	1.72	N/A
930	1.63	-1.67	N/A
931	1.63	1.88	N/A
932	1.63	1.39	hypothetical protein POPTR_0008s01370g
933	1.64	-1.30	N/A
934	1.64	1.09	N/A
935	1.64	-1.25	hypothetical protein POPTR_0004s18970g
936	1.65	-1.18	PREDICTED: nudix hydrolase 8-like isoform X3 [*Populus euphratica*]
937	1.65	-1.52	N/A
938	1.65	-1.63	N/A
939	1.65	1.16	N/A
940	1.65	-1.38	N/A
941	1.65	1.09	N/A
942	1.66	-1.41	unknown
943	1.66	1.46	hypothetical protein POPTR_0004s01220g
944	1.66	1.06	hypothetical protein POPTR_0004s01220g
945	1.67	-2.69	hypothetical protein POPTR_0002s07630g
946	1.67	-1.12	hypothetical protein POPTR_0011s04240g
947	1.68	1.37	N/A
948	1.68	-1.15	N/A
949	1.68	1.00	dynamin family protein
950	1.68	-1.55	N/A
951	1.68	1.41	N/A
952	1.68	-1.10	hypothetical protein POPTR_0006s04930g
953	1.69	2.60	hypothetical protein POPTR_0006s27220g
954	1.69	1.53	hypothetical protein POPTR_0004s20680g
955	1.70	1.08	N/A

956	1.70	1.04	N/A
957	1.70	3.73	N/A
958	1.70	2.60	hypothetical protein POPTR_0017s07260g
959	1.70	1.93	N/A
960	1.70	1.39	1-aminocyclopropane-1-carboxylate synthase family protein
961	1.70	1.34	N/A
962	1.70	1.06	N/A
963	1.70	-3.54	N/A
964	1.71	-1.17	N/A
965	1.71	3.49	hypothetical protein POPTR_0001s46760g
966	1.71	-1.51	N/A
967	1.72	2.19	unknown
968	1.74	-1.15	N/A
969	1.74	1.04	N/A
970	1.74	1.19	N/A
971	1.74	1.24	hypothetical protein POPTR_0014s01850g
972	1.75	1.16	N/A
973	1.75	-1.50	PREDICTED: uncharacterized protein LOC105136171 [*Populus euphratica*]
974	1.75	-1.78	N/A
975	1.75	1.38	N/A
976	1.76	1.56	N/A
977	1.76	-1.43	hypothetical protein POPTR_0014s02900g
978	1.76	1.12	beta-amylase family protein
979	1.76	-2.76	hypothetical protein POPTR_0019s10710g
980	1.76	0.99	hypothetical protein POPTR_0006s23780g
981	1.77	1.67	PREDICTED: two-component response regulator-like APRR5 isoform X1 [*Populus euphratica*]
982	1.77	2.24	N/A
983	1.78	2.05	1-aminocyclopropane-1-carboxylate synthase family protein
984	1.79	0.99	PREDICTED: two-component response regulator-like APRR5 isoform X1 [*Populus euphratica*]
985	1.79	1.06	hypothetical protein POPTR_0018s08320g
986	1.80	-1.42	PREDICTED: two-component response regulator-like APRR5 isoform X1 [*Populus euphratica*]
987	1.81	0.99	hypothetical protein POPTR_0014s16610g
988	1.81	1.50	PREDICTED: two-component response regulator-like APRR5 isoform X1 [*Populus euphratica*]
989	1.82	1.26	PREDICTED: two-component response regulator-like APRR5 isoform X1 [*Populus euphratica*]
990	1.83	1.74	PREDICTED: two-component response regulator-like APRR5 isoform X1 [*Populus euphratica*]
991	1.83	1.35	PREDICTED: two-component response regulator-like APRR5 isoform X1 [*Populus euphratica*]
992	1.83	1.73	hypothetical protein POPTR_0017s07510g
993	1.84	1.10	hypothetical protein POPTR_0017s07280g
994	1.84	0.98	myb family transcription factor family protein
995	1.84	-1.10	N/A
996	1.84	2.15	hypothetical protein POPTR_0014s02900g
997	1.85	-3.07	N/A
998	1.85	-2.16	N/A
999	1.86	1.22	hypothetical protein POPTR_0014s07840g
1000	1.88	1.39	hypothetical protein POPTR_0002s07130g
1001	1.89	-1.17	N/A
1002	1.89	-2.80	N/A
1003	1.89	-3.11	hypothetical protein POPTR_0005s10580g

1004	1.90	1.53	N/A
1005	1.90	-2.27	hypothetical protein POPTR_0008s02970g
1006	1.90	1.36	hypothetical protein POPTR_0008s02970g
1007	1.90	1.99	hypothetical protein POPTR_0008s02970g
1008	1.91	-1.41	N/A
1009	1.92	1.13	N/A
1010	1.94	1.62	N/A
1011	1.94	1.35	N/A
1012	1.94	-1.60	hypothetical protein POPTR_0005s10580g
1013	1.94	1.45	hypothetical protein POPTR_0005s10580g
1014	1.94	-2.63	N/A
1015	1.95	-2.42	pectinesterase family protein
1016	1.95	1.32	N/A
1017	1.97	1.60	N/A
1018	1.97	-2.86	N/A
1019	1.97	2.55	N/A
1020	1.99	1.34	hypothetical protein POPTR_0009s08930g
1021	2.01	-1.23	N/A
1022	2.02	-1.51	hypothetical protein POPTR_0001s46680g
1023	2.02	-1.20	N/A
1024	2.02	1.79	N/A
1025	2.03	-2.05	hypothetical protein POPTR_0012s02850g
1026	2.03	-1.37	N/A
1027	2.04	1.06	N/A
1028	2.05	-3.07	N/A
1029	2.08	1.03	hypothetical protein POPTR_0011s14600g
1030	2.08	1.27	N/A
1031	2.09	-1.53	hypothetical protein POPTR_0006s08740g
1032	2.10	1.01	N/A
1033	2.11	1.05	no apical meristem family protein

1034	2.11	1.69	hypothetical protein POPTR_0011s00400g
1035	2.11	1.03	N/A
1036	2.12	1.19	hypothetical protein POPTR_0002s16970g
1037	2.12	2.44	N/A
1038	2.15	2.25	N/A
1039	2.16	-1.33	hypothetical protein POPTR_0014s17550g
1040	2.17	2.52	N/A
1041	2.19	-	N/A
1042	2.21	-1.23	N/A
1043	2.21	4.19	cytidine
1044	2.21	1.05	hypothetical protein POPTR_0004s21320g
1045	2.23	-1.51	N/A
1046	2.28	1.04	N/A
1047	2.28	1.43	N/A
1048	2.28	3.79	PREDICTED: histone H1.1-like [*Populus euphratica*]
1049	2.30	-1.32	N/A
1050	2.33	1.37	N/A
1051	2.34	1.55	hypothetical protein POPTR_0015s00280g
1052	2.37	-1.59	N/A
1053	2.37	1.26	N/A
1054	2.37	-3.85	N/A
1055	2.38	1.12	hypothetical protein POPTR_0006s14000g
1056	2.42	1.39	N/A
1057	2.48	-2.16	N/A
1058	2.51	2.74	N/A
1059	2.52	1.48	N/A
1060	2.55	-1.58	N/A
1061	2.60	1.77	N/A
1062	2.63	-1.74	hypothetical protein POPTR_0018s01890g
1063	2.64	-1.19	hypothetical protein POPTR_0001s46680g

1064	2.69	-3.18	N/A
1065	2.73	1.59	N/A
1066	2.74	1.14	hypothetical protein POPTR_0007s03640g
1067	2.76	-	N/A
1068	2.77	-2.18	N/A
1069	2.77	-1.37	N/A
1070	2.77	1.26	PREDICTED: neurogenic locus notch homolog protein 2-like [*Populus euphratica*]
1071	2.81	1.14	hypothetical protein POPTR_0013s05400g, partial
1072	2.83	1.13	N/A
1073	2.89	3.07	N/A
1074	2.90	-	N/A
1075	2.91	1.18	hypothetical protein POPTR_0002s16910g
1076	2.95	-1.23	N/A
1077	2.96	2.08	N/A
1078	2.98	1.71	trypsin inhibitor family protein
1079	3.02	1.42	N/A
1080	3.04	-1.05	unknown
1081	3.10	-1.01	N/A
1082	3.13	1.01	N/A
1083	3.24	-1.38	N/A
1084	3.30	-2.08	N/A
1085	3.32	-1.17	N/A
1086	3.33	2.39	hypothetical protein POPTR_0006s23480g
1087	3.42	1.31	N/A
1088	3.44	-	N/A
1089	3.44	-1.75	N/A
1090	3.44	1.60	N/A
1091	3.47	-	hypothetical protein POPTR_0770s00220g
1092	3.50	-1.53	N/A
1093	3.50	1.02	hypothetical protein POPTR_0770s00220g

1094	3.77	-1.14	N/A
1095	3.84	1.75	N/A
1096	3.99	-	N/A
1097	4.04	1.31	N/A
1098	4.12	-1.25	N/A
1099	4.18	-1.36	hypothetical protein POPTR_0010s08600g
1100	4.20	2.14	N/A
1101	4.27	1.47	hypothetical protein POPTR_0011s11160g
1102	4.41	-	N/A
1103	4.46	-1.22	N/A
1104	4.86	2.17	N/A
1105	-	-5.56	N/A
1106	-	-2.59	N/A
1107	-	-2.44	N/A
1108	-	-2.05	hypothetical protein POPTR_0001s27010g
1109	-	-1.95	N/A
1110	-	-1.90	N/A
1111	-	-1.86	N/A
1112	-	-1.80	hypothetical protein POPTR_0002s04250g
1113	-	-1.50	N/A
1114	-	-1.40	N/A
1115	-	-1.38	hypothetical protein POPTR_0001s27420g
1116	-	-1.32	N/A
1117	-	-1.31	PREDICTED: MATE efflux family protein 5-like [*Populus euphratica*]
1118	-	-1.28	N/A
1119	-	-1.25	N/A
1120	-	-1.23	hypothetical protein POPTR_0011s14830g
1121	-	-1.23	N/A
1122	-	-1.04	N/A
1123	-	1.01	hypothetical protein POPTR_0010s21830g

1124	-	1.02	N/A
1125	-	1.04	N/A
1126	-	1.06	hypothetical protein POPTR_0010s14370g
1127	-	1.06	N/A
1128	-	1.09	hypothetical protein POPTR_0001s16970g
1129	-	1.10	hypothetical protein POPTR_0003s09660g
1130	-	1.10	N/A
1131	-	1.11	hypothetical protein POPTR_0013s14870g
1132	-	1.17	N/A
1133	-	1.22	hypothetical protein POPTR_0010s05100g
1134	-	1.24	hypothetical protein POPTR_0011s00430g
1135	-	1.27	VQ motif-containing family protein
1136	-	1.28	N/A
1137	-	1.29	hypothetical protein POPTR_0010s20480g
1138	-	1.29	N/A
1139	-	1.37	N/A
1140	-	1.45	hypothetical protein POPTR_0008s07220g
1141	-	1.47	hypothetical protein POPTR_0001s16970g
1142	-	1.51	hypothetical protein POPTR_0019s03120g
1143	-	1.57	N/A
1144	-	1.57	PREDICTED: kinesin-like protein klp-3 [*Populus euphratica*]
1145	-	1.58	chloroplast nucleoid DNA-binding family protein
1146	-	1.59	N/A
1147	-	1.65	N/A
1148	-	1.66	N/A
1149	-	2.12	N/A
1150	-	2.14	N/A
1151	-	3.32	N/A
1152	-1.46	1.86	NAC (No Apical Meristem) domain transcriptional regulator superfamily protein
1153	1.56	-1.23	nodulin MtN21 /EamA-like transporter family protein

1154	1.54	1.10	nuclear factor Y, subunit A6
1155	1.64	-1.28	nudix hydrolase homolog 8
1156	-1.42	1.40	O-Glycosyl hydrolases family 17 protein
1157	-1.25	-2.15	OPC-8:0 CoA ligase1
1158	1.00	1.32	Outer arm dynein light chain 1 protein
1159	1.08	-4.47	Pathogenesis-related thaumatin superfamily protein
1160	1.14	1.82	Pathogenesis-related thaumatin superfamily protein
1161	-	1.33	Pentatricopeptide repeat (PPR) superfamily protein
1162	1.11	-2.56	Peroxidase superfamily protein
1163	1.43	1.50	Peroxidase superfamily protein
1164	4.63	-	Peroxidase superfamily protein
1165	1.97	-1.07	P-glycoprotein 18
1166	1.06	-1.90	phosphate transporter 3
1167	1.70	2.11	Phosphate-responsive 1 family protein
1168	1.97	1.43	Phosphate-responsive 1 family protein
1169	1.24	1.38	Phosphorylase superfamily protein
1170	-1.45	1.24	Plant invertase/pectin methylesterase inhibitor superfamily
1171	1.28	-1.30	Plant protein of unknown function (DUF247)
1172	1.15	1.59	Plant protein of unknown function (DUF868)
1173	1.06	2.21	plant U-box 29
1174	-1.46	1.04	PLC-like phosphodiesterases superfamily protein
1175	1.10	2.33	P-loop containing nucleoside triphosphate hydrolases superfamily protein
1176	0.95	1.20	Protein kinase superfamily protein
1177	1.08	1.44	Protein kinase superfamily protein
1178	-1.53	1.38	Protein of unknown function (DUF1677)
1179	2.06	2.02	Protein of unknown function (DUF3049)
1180	-1.13	1.77	Protein of unknown function (DUF567)
1181	1.32	-1.22	Protein of unknown function (DUF668)
1182	1.34	1.42	Protein of unknown function (DUF761)
1183	1.45	2.62	Protein of unknown function (DUF761)

1184	-1.46	1.89	Protein of unknown function, DUF538
1185	1.02	1.56	proton gradient regulation 5
1186	1.80	-1.42	pseudo-response regulator 5
1187	1.81	1.24	pseudo-response regulator 5
1188	1.12	0.99	purple acid phosphatase 17
1189	1.16	-1.69	purple acid phosphatase 17
1190	1.52	-1.99	purple acid phosphatase 17
1191	1.37	-1.80	purple acid phosphatase 22
1192	1.17	1.73	RAD-like 6
1193	1.19	1.19	RAD-like 6
1194	1.03	-2.93	receptor like protein 43
1195	-2.21	2.61	Regulator of chromosome condensation (RCC1) family protein
1196	-1.48	-1.31	RING/FYVE/PHD zinc finger superfamily protein
1197	-1.44	-1.43	RING/FYVE/PHD zinc finger superfamily protein
1198	-1.41	0.99	RING/U-box superfamily protein
1199	-2.06	1.05	S-adenosyl-L-methionine-dependent methyltransferases superfamily protein
1200	1.44	1.03	S-adenosyl-L-methionine-dependent methyltransferases superfamily protein
1201	-1.29	1.34	salt tolerance homolog2
1202	1.02	1.08	salt tolerance zinc finger
1203	-1.64	0.98	SAUR-like auxin-responsive protein family
1204	-2.06	1.19	SC35-like splicing factor 33
1205	-1.67	1.69	SC35-like splicing factor 33
1206	-1.60	1.62	SC35-like splicing factor 33
1207	4.48	-1.49	serine protease inhibitor, Kazal-type family protein
1208	5.94	-	serine protease inhibitor, Kazal-type family protein
1209	1.28	2.22	sigma factor E
1210	1.20	1.55	S-locus lectin protein kinase family protein
1211	1.15	1.21	solanesyl diphosphate synthase 1
1212	1.37	1.39	SPX domain gene 2
1213	1.08	1.14	Subtilase family protein

1214	-1.15	-1.55	T-complex protein 11
1215	1.28	-1.16	thioredoxin H-type 8
1216	1.00	1.64	Transducin/WD40 repeat-like superfamily protein
1217	-	1.24	UDP-glycosyltransferase 73B4
1218	-1.88	1.03	UDP-Glycosyltransferase superfamily protein
1219	0.99	-1.29	UDP-Glycosyltransferase superfamily protein
1220	1.02	1.94	UDP-Glycosyltransferase superfamily protein
1221	1.04	1.43	wall-associated kinase 2
1222	-1.23	1.84	Wound-responsive family protein
1223	-1.12	1.51	Wound-responsive family protein
1224	1.47	1.79	WRKY DNA-binding protein 28
1225	1.55	-5.86	WRKY DNA-binding protein 75
1226	0.97	-0.07	WRKY family transcription factor
1227	1.14	4.00	WRKY family transcription factor
1228	2.15	-2.02	zinc knuckle (CCHC-type) family protein

N/A indicates no annotation acquired.

Table S2. Expression of TFs at 12 hpi and 4 dpi.

No.	TF Family	TF Number	Time Points	Changed TFs		Unchanged TFs	Undetected
				upregulated	downregulated		
**1**	**AP2**	**59**	**12 hpi**	**5**	5	43	6
			4 dpi	17	5	26	11
2	ARF	98	12 hpi	2	0	96	0
			4 dpi	13	8	77	0
3	ARR-B	63	12 hpi	0	0	59	4
			4 dpi	20	0	39	4
4	B3	146	12 hpi	20	12	77	37
			4 dpi	54	18	54	20
5	BBR-BPC	33	12 hpi	0	0	33	0
			4 dpi	5	2	26	0
6	BES1	32	12 hpi	0	1	30	1
			4 dpi	4	1	26	1
7	C2H2	204	12 hpi	20	7	144	33
			4 dpi	39	21	114	30
8	C3H	119	12 hpi	1	6	112	0
			4 dpi	5	19	95	0
9	CAMTA	18	12 hpi	0	0	18	0
			4 dpi	0	0	18	0
10	CO-like	31	12 hpi	0	1	30	0
			4 dpi	5	0	26	0
11	CPP	25	12 hpi	4	4	15	2
			4 dpi	9	4	11	1
12	DBB	34	12 hpi	1	1	32	0
			4 dpi	25	3	6	0
13	Dof	62	12 hpi	2	5	53	2
			4 dpi	9	12	38	3
14	E2F/DP	16	12 hpi	0	0	16	0
			4 dpi	3	0	13	0
15	EIL	19	12 hpi	0	0	19	0
			4 dpi	0	2	17	0
16	ERF	209	12 hpi	26	18	133	32
			4 dpi	73	32	71	33
17	FAR1	111	12 hpi	0	7	103	1
			4 dpi	11	4	94	2
18	G2-like	146	12 hpi	15	10	115	6
			4 dpi	39	6	94	7
19	GATA	76	12 hpi	2	2	70	2
			4 dpi	18	5	52	1
20	GRAS	151	12 hpi	6	4	118	23
			4 dpi	44	13	73	21
21	GRF	26	12 hpi	2	4	18	2
			4 dpi	11	2	11	2
22	GeBP	6	12 hpi	0	1	5	0
			4 dpi	0	0	6	0
23	HB-PHD	10	12 hpi	0	0	10	0
			4 dpi	2	0	8	0
24	HB-other	34	12 hpi	0	0	34	0
			4 dpi	0	0	34	0
25	HD-ZIP	114	12 hpi	3	7	101	3
			4 dpi	29	8	73	4
26	HRT-like	5	12 hpi	0	0	5	0
			4 dpi	0	0	5	0
27	HSF	47	12 hpi	1	10	36	0

			4 dpi	8	5	34	0
28	LBD	70	12 hpi	9	22	15	24
			4 dpi	21	16	12	21
29	LFY	1	12 hpi	0	0	0	1
			4 dpi	0	1	0	0
30	LSD	15	12 hpi	0	0	15	0
			4 dpi	4	0	11	0
31	M-type	58	12 hpi	8	7	7	36
			4 dpi	14	9	5	30
32	MIKC	123	12 hpi	22	5	84	12
			4 dpi	31	21	53	18
33	MYB	266	12 hpi	36	19	175	36
			4 dpi	99	42	90	35
34	MYB_related	213	12 hpi	21	3	184	5
			4 dpi	32	13	162	6
35	NAC	289	12 hpi	35	30	190	34
			4 dpi	85	30	135	39
36	NF-X1	4	12 hpi	0	0	4	0
			4 dpi	0	0	4	0
37	NF-YA	57	12 hpi	4	0	52	1
			4 dpi	4	10	42	1
38	NF-YB	38	12 hpi	1	2	30	5
			4 dpi	6	1	25	6
39	NF-YC	27	12 hpi	0	0	22	5
			4 dpi	2	2	18	5
40	NZZ/SPL	4	12 hpi	0	0	2	2
			4 dpi	0	0	0	4
41	Nin-like	30	12 hpi	0	1	25	4
			4 dpi	3	5	18	4
42	RAV	5	12 hpi	0	0	4	1
			4 dpi	4	0	0	1
43	S1Fa-like	2	12 hpi	0	0	2	0
			4 dpi	0	0	2	0
44	SAP	1	12 hpi	0	0	1	0
			4 dpi	0	0	1	0
45	SBP	68	12 hpi	6	1	60	1
			4 dpi	13	8	46	1
46	SRS	21	12 hpi	1	2	15	3
			4 dpi	6	6	6	3
47	STAT	4	12 hpi	0	0	4	0
			4 dpi	0	0	4	0
48	TALE	80	12 hpi	2	1	70	7
			4 dpi	13	5	53	9
49	TCP	60	12 hpi	1	4	52	3
			4 dpi	8	3	44	5
50	Trihelix	78	12 hpi	2	5	70	1
			4 dpi	10	2	65	1
51	VOZ	8	12 hpi	0	0	8	0
			4 dpi	2	0	6	0
52	WOX	26	12 hpi	6	2	9	9
			4 dpi	6	3	8	9
53	WRKY	185	12 hpi	25	4	154	2
			4 dpi	72	8	105	0
54	Whirly	6	12 hpi	0	0	6	0
			4 dpi	0	1	5	0
55	YABBY	35	12 hpi	0	1	31	3
			4 dpi	0	6	26	3
56	ZF-HD	26	12 hpi	2	3	18	3

			4 dpi	10	4	10	2
57	bHLH	379	12 hpi	15	28	293	43
			4 dpi	106	42	202	29
58	bZIP	215	12 hpi	4	5	196	10
			4 dpi	28	21	155	11
**Sum**	**-**	**4, 288**	**12 hpi**	**310**	**250**	**3, 323**	**405**
			**4 dpi**	**1, 022**	**429**	**2, 454**	**383**

Table S3. The network of 5 TF families that regulate potential target genes with their corresponding TF-binding motifs.

ID of homologous genes of *Populus trichocarpa*	Log_2	NAC	MYB	ERF	AP2	WRKY	ATG	ATG_Synonyms
Potri.001G150000	2.0	0	0	0	1	0	AT5G04560	DME
Potri.001G392300	2.0	1	0	0	0	0	AT3G50930	BCS1
Potri.002G043300	2.0	0	1	1	1	1	AT5G44210	ATERF-9,ATERF9,ERF9
Potri.003G220200	2.0	0	1	0	0	0	AT5G19790	RAP2.11
Potri.004G161900	2.0	0	0	0	1	0	AT4G39010	AtGH9B18,GH9B18
Potri.004G162200	2.0	0	0	0	1	0	AT4G39010	AtGH9B18,GH9B18
Potri.005G066100	2.0	0	1	1	1	1	AT5G25830	GATA12
Potri.009G100500	2.0	0	1	0	1	0	AT4G34980	SLP2
Potri.010G057900	2.0	1	0	0	0	0	AT3G50930	BCS1
Potri.010G123900	2.0	1	0	0	0	0	AT1G68460	ATIPT1,IPT1
Potri.015G046800	2.0	1	0	0	0	0	AT3G18400	anac058,NAC058
Potri.016G063900	2.0	0	1	0	0	0	AT2G38110	ATGPAT6,GPAT6
Potri.018G049300	2.0	1	0	0	0	0	AT1G26870	ANAC009,FEZ
Potri.019G067400	2.0	1	0	0	1	0	AT5G25810	tny
Potri.002G163700	1.7	0	1	0	0	0	AT3G61510	ACS1,AT-ACS1
Potri.011G057000	1.7	0	1	1	1	1	AT5G44210	ATERF-9,ATERF9,ERF9
Potri.010G147700	1.5	1	0	0	0	0	AT5G49520	ATWRKY48,WRKY48

Potri.013G054200	1.5	0	0	0	1	0	AT5G18270	ANAC087
Potri.T004800	1.5	0	1	0	0	0	AT5G14930	SAG101
Potri.001G364600	1.4	1	0	0	0	0	AT3G26300	CYP71B34
Potri.001G362600	1.4	1	0	0	0	0	AT3G26300	CYP71B34
Potri.007G045300	1.3	0	0	0	1	0	AT5G67100	ICU2
Potri.001G363100	1.3	1	0	0	0	0	AT3G26300	CYP71B34
Potri.004G051700	1.2	0	0	1	0	0	AT4G17500	ATERF-1,ERF-1
Potri.001G138800	1.2	0	1	1	0	1	AT1G32320	ATMKK10,MKK10
Potri.008G103300	1.2	1	0	0	0	0	AT5G49520	ATWRKY48,WRKY48
Potri.T004600	1.2	0	1	0	0	0	AT5G14930	SAG101
Potri.017G013700	1.2	0	1	1	1	1	AT5G44210	ATERF-9,ATERF9,ERF9
Potri.004G101400	1.2	0	1	1	0	1	AT5G16080	AtCXE17,CXE17
Potri.001G365300	1.2	1	0	0	0	0	AT3G26300	CYP71B34
Potri.001G365400	1.1	1	0	0	0	0	AT3G26300	CYP71B34
Potri.005G054300	1.1	1	0	0	1	1	AT3G04720	HEL,PR-4,PR4
Potri.006G097800	1.1	0	1	0	0	0	AT2G38110	ATGPAT6,GPAT6
Potri.007G100600	1.1	0	0	0	0	1	AT3G52430	ATPAD4,PAD4
Potri.001G364900	1.1	1	0	0	0	0	AT3G26300	CYP71B34
Potri.001G144400	1.1	1	0	0	0	0	AT4G17980	anac071,NAC071
Potri.019G031600	1.1	0	0	0	1	0	AT5G18270	ANAC087

Potri.002G224100	1.1	0	1	0	0	0	AT1G05010	ACO4,EAT1,EFE
Potri.008G025500	1.0	0	0	0	1	0	AT5G04660	CYP77A4
Potri.011G130800	1.0	1	0	0	0	0	AT3G26300	CYP71B34
Potri.001G362800	1.0	1	0	0	0	0	AT3G26300	CYP71B34
Potri.001G363900	1.0	1	0	0	0	0	AT3G26300	CYP71B34
Potri.003G089800	1.0	1	0	0	0	0	AT4G17980	anac071,NAC071
Potri.019G099900	1.0	1	0	0	0	0	AT4G17980	anac071,NAC071
Potri.T127100	1.0	1	0	0	0	0	AT3G26300	CYP71B34
Potri.007G082900	-1.0	1	0	0	0	0	AT3G26300	CYP71B34
Potri.005G112800	-1.0	0	1	0	1	1	AT4G36020	CSDP1
Potri.014G090500	-1.0	1	0	0	1	0	AT2G46110	KPHMT1,PANB1
Potri.007G084800	-1.0	1	0	0	0	0	AT3G26300	CYP71B34
Potri.013G041600	-1.0	1	0	0	1	1	AT3G04720	HEL,PR-4,PR4
Potri.014G103700	-1.1	0	0	0	0	1	AT1G32640	ATMYC2,JAI1,JIN1,MYC2,RD22BP1,ZBF1
Potri.006G236200	-1.1	0	1	1	1	1	AT3G16500	IAA26,PAP1
Potri.001G332300	-1.1	1	0	0	0	0	AT5G14180	MPL1
Potri.010G127600	-1.1	0	1	1	0	1	AT5G16080	AtCXE17,CXE17
Potri.002G151900	-1.2	0	1	1	1	1	AT4G00230	XSP1
Potri.017G053100	-1.3	0	1	1	0	0	AT1G02170	AMC1,ATMC1,ATMCPB1,LOL3,MCP1B
Potri.009G123900	-1.3	0	0	0	1	0	AT4G39010	AtGH9B18,GH9B18

Potri.008G101600	-1.4	1	0	0	0	0	AT1G47990	ATGA2OX4,GA2OX4
Potri.016G007700	-1.4	1	0	0	0	0	AT4G13290	CYP71A19
Potri.007G044800	-1.4	0	0	0	1	0	AT5G67080	MAPKKK19
Potri.T117300	-1.4	0	1	0	0	0	AT5G14930	SAG101
Potri.001G002200	-1.5	0	1	1	0	1	AT4G26330	ATSBT3.18,UNE17
Potri.002G176900	-1.5	0	0	0	0	1	AT1G32640	ATMYC2,JAI1,JIN1,MYC2,RD22BP1,ZBF1
Potri.014G159800	-1.6	1	0	0	0	0	AT5G14180	MPL1
Potri.012G128200	-1.7	0	1	1	1	1	AT1G02000	GAE2
Potri.014G159900	-1.7	1	0	0	0	0	AT5G14180	MPL1
Potri.015G133800	-1.8	1	0	0	0	0	AT5G51750	ATSBT1.3,SBT1.3
Potri.002G082400	-1.9	1	0	0	1	0	AT1G44350	ILL6
Potri.001G083200	-2.0	0	1	0	0	0	AT1G02800	ATCEL2,CEL2
Potri.001G083500	-2.0	0	0	0	0	1	AT1G32640	ATMYC2,JAI1,JIN1,MYC2,RD22BP1,ZBF1
Potri.001G453100	-2.0	0	1	0	0	0	AT5G19790	RAP2.11
Potri.002G060900	-2.0	1	0	0	0	0	AT5G09970	CYP78A7
Potri.004G139200	-2.0	0	0	0	0	1	AT1G70170	MMP
Potri.004G161600	-2.0	0	1	0	1	1	AT4G36220	CYP84A1,FAH1
Potri.005G115200	-2.0	1	0	0	0	0	AT3G50930	BCS1
Potri.007G044900	-2.0	0	0	0	1	0	AT5G67080	MAPKKK19
Potri.008G033300	-2.0	0	0	0	0	1	AT5G19040	ATIPT5,IPT5

Potri.008G166500	-2.0	0	0	0	1	1	AT1G08650	ATPPCK1,PPCK1
Potri.011G111600	-2.0	0	0	0	0	1	AT1G70170	MMP
Potri.011G148900	-2.0	0	1	0	0	0	AT5G19790	RAP2.11
Potri.014G159700	-2.0	1	0	0	0	0	AT5G14180	MPL1
Potri.017G052600	-2.0	0	1	1	0	0	AT1G02170	AMC1,ATMC1,ATMCPB1,LOL3,MCP1B
Potri.017G087800	-2.0	0	1	0	0	0	AT5G19790	RAP2.11
Potri.005G040200	-2.2	0	1	1	0	1	AT2G48010	RKF3
Potri.005G084500	-3.6	1	0	0	0	0	AT5G09970	CYP78A7
Potri.003G147300	-3.6	0	0	0	0	1	AT1G32640	ATMYC2,JAI1,JIN1,MYC2,RD22BP1,ZBF1

Table S4. KEGG reference pathways.

No.	KO	Reference Pathways	Pathway Number
1	ko01100	Metabolic pathways	20
2	ko01110	Biosynthesis of secondary metabolites	14
3	ko04110	Cell cycle	5
4	ko04111	Cell cycle - yeast	5
5	ko04113	Meiosis - yeast	4
6	ko04075	Plant hormone signal transduction	3
7	ko00562	Inositol phosphate metabolism	2
8	ko00010	Glycolysis / Gluconeogenesis	2
9	ko00904	Diterpenoid biosynthesis	2
10	ko04114	Oocyte meiosis	2
11	ko00040	Pentose and glucuronate interconversions	2
12	ko01130	Biosynthesis of antibiotics	2
13	ko04020	Calcium signaling pathway	2
14	ko00561	Glycerolipid metabolism	2
15	ko00940	Phenylpropanoid biosynthesis	2
16	ko00250	Alanine, aspartate and glutamate metabolism	2
17	ko00500	Starch and sucrose metabolism	2
18	ko00564	Glycerophospholipid metabolism	2
19	ko00906	Carotenoid biosynthesis	2
20	ko01120	Microbial metabolism in diverse environments	2
21	ko04914	Progesterone-mediated oocyte maturation	2
22	ko00520	Amino sugar and nucleotide sugar metabolism	2
23	ko00941	Flavonoid biosynthesis	2
24	ko04919	Thyroid hormone signaling pathway	2
25	ko05166	HTLV-I infection	1
26	ko01040	Biosynthesis of unsaturated fatty acids	1
27	ko04022	cGMP-PKG signaling pathway	1
28	ko04924	Renin secretion	1
29	ko04724	Glutamatergic synapse	1
30	ko00680	Methane metabolism	1
31	ko04650	Natural killer cell mediated cytotoxicity	1
32	ko04310	Wnt signaling pathway	1
33	ko00604	Glycosphingolipid biosynthesis - ganglio series	1
34	ko03030	DNA replication	1
35	ko00860	Porphyrin and chlorophyll metabolism	1
36	ko00410	beta-Alanine metabolism	1
37	ko01230	Biosynthesis of amino acids	1
38	ko00340	Histidine metabolism	1
39	ko00620	Pyruvate metabolism	1
40	ko00903	Limonene and pinene degradation	1
41	ko00785	Lipoic acid metabolism	1
42	ko00710	Carbon fixation in photosynthetic organisms	1
43	ko05152	Tuberculosis	1
44	ko04370	VEGF signaling pathway	1
45	ko04712	Circadian rhythm - plant	1
46	ko04933	AGE-RAGE signaling pathway in diabetic complications	1
47	ko04922	Glucagon signaling pathway	1
48	ko05031	Amphetamine addiction	1
49	ko00052	Galactose metabolism	1
50	ko04662	B cell receptor signaling pathway	1
51	ko00380	Tryptophan metabolism	1
52	ko05203	Viral carcinogenesis	1
53	ko05161	Hepatitis B	1
54	ko00565	Ether lipid metabolism	1
55	ko04921	Oxytocin signaling pathway	1
56	ko04910	Insulin signaling pathway	1
57	ko04152	AMPK signaling pathway	1

58	ko00280	Valine, leucine and isoleucine degradation	1
59	ko04626	Plant-pathogen interaction	1
60	ko04720	Long-term potentiation	1
61	ko04380	Osteoclast differentiation	1
62	ko05010	Alzheimer's disease	1
63	ko00625	Chloroalkane and chloroalkene degradation	1
64	ko00760	Nicotinate and nicotinamide metabolism	1
65	ko00051	Fructose and mannose metabolism	1
66	ko04070	Phosphatidylinositol signaling system	1
67	ko00030	Pentose phosphate pathway	1
68	ko00270	Cysteine and methionine metabolism	1
69	ko03410	Base excision repair	1
70	ko04010	MAPK signaling pathway	1
71	ko01200	Carbon metabolism	1
72	ko05014	Amyotrophic lateral sclerosis (ALS)	1
73	ko00310	Lysine degradation	1
74	ko00511	Other glycan degradation	1
75	ko05169	Epstein-Barr virus infection	1
76	ko00603	Glycosphingolipid biosynthesis - globo series	1
77	ko00053	Ascorbate and aldarate metabolism	1
78	ko04360	Axon guidance	1
79	ko04142	Lysosome	1
80	ko00071	Fatty acid degradation	1
81	ko00531	Glycosaminoglycan degradation	1
82	ko04660	T cell receptor signaling pathway	1
83	ko00330	Arginine and proline metabolism	1
84	ko01212	Fatty acid metabolism	1

Table S5. Primers used in this study.

Sequence	ID of homologous genes of *Populus trichocarpa*	No.	F primer	R primer
Mlp ITS			TGAGCGACTTTAATGTGACTC	TGTAAATCAAAGTTGCCTTTGCG
'Robusta' ITS			GAAGTAGCACTTGCTGCATAG	GTCAAAATCTTGGGAGTCAAAG
NAC	Potri.017G082000	1	GGGTTTTGGAAGGCGACTG	CTTTGGTGGTGGTGGTTCAG
	Potri.014G104800	2	CTGCTGCTGGGTTTTGGAAG	GGTATCACTGGTGCTATCGTC
	Potri.002G057200	3	CTCTCGCGTGATTATACATGGT	CGCCGCAGATAGAACCCA
	Potri.011G046700	4	GGGTATTGGAAAGCTACTGGA	CCAGGAGGTTTTGAGCTAGG
	Potri.001G080900	5	GCATCAGACCTAATAGGGTCAC	GCGAAATTCGTGCATCATCC
	Potri.004G038000	6	GGTATTGGAAAGCTACCGGG	GGGGCTTTGGGCTAGAATTG
	Potri.007G014400	7	AGATGCCGGATCGGATATGA	ACAAGGGTTTTCCTCATGCC
	Potri.017G016700	8	GAAGTCTGATACCCGCAAGG	TTGATGGCCGATGGAAGAAG
	Potri.002G178700	9	AAGAAATATCCAGCCGGCAC	GATTTTTGTCCATGTGGGGC
	Potri.003G046700	10	GATCCTTGGGAACTTCCTGC	GATCATCCGGTCAGCTCCT
	Potri.003G149700	11	TGTAGTGCTGGAGAAAGGGA	AGGCCAATGCAGTAATGGAG
	Potri.011G153300	12	GGGACAAGAACAAATCGTGC	CGGATTTCTGACCATGTGGA
	Potri.019G083600	13	GCTGGATTTTGGAAGGCAAC	GGGGTCCATGTTCAGATGTT
	Potri.003G089800	14	CTCAAGCTGGCTACTGGAAG	GCATTACCCAGTCTGTCCTG
	Potri.019G099900	15	GGTTCATGGCTAGCTACAGG	AGAGAAGTACTCAGGTTGGGT
	Potri.019G031600	16	TCGTGCTACAGAATCAGGGT	TTGGGAGCTCTCCCTTTGTA
	Potri.001G144400	17	GGGCCACTAAATCTGGCTAC	TCATCGCAAAGGCGATACTC
MYB	Potri.001G169600	1	GAGGACAATCGCATCCAAAGC	CGGTAATCTTCCGGCAATCAG
	Potri.001G258700	2	CATCGTTGGAAACCTCCATGC	GCCCATACAACCCATGATCAGA
	Potri.006G123400	3	TGAGCACTAGCTCTTTCGCT	TGCAACTCCCATCAGACGAG
	Potri.006G170800	4	CCCTGAAAGAAAGCTTTAGCCC	ATTCTCCAAGCCCACTACGC
	Potri.006G221500	5	CAGATTCTCACAGCATCCTCCA	TCAGAGAAGCTCATCTGCCC
	Potri.007G093900	6	CTCAACCTCCTGCAGCTTCT	AGCCGGTAAATCCAACTGGG
	Potri.008G070900	7	CCACGGCAATAAGTGGGCTA	ACTCAGACACAACCGACACC

	Potri.008G148400	8	ACGCAGTTCAAAGCAGTAGGA	TGCTCCGGTTCAGGAAATCT
	Potri.008G166700	9	GTGATCACTCCTCAGTCACGG	GGCATGCCAGAATTCTCAACCA
	Potri.010G093000	10	AGGTCTCCATTGATGCAGTTCA	TCCGATTCAGGAAATCTTGGAGA
	Potri.012G039400	11	TGGTCGCACCGATAATGCAG	ATCCATGTGCATGTGATCTTGT
	Potri.013G130900	12	TGATGAGGGTGGTGAATTCCAG	TGTGTGGTCCATCGCACTC
	Potri.015G082700	13	CCCTGCCGGTATTGATCTTACT	ATCGGAGAAATCCGTCCCAC
	Potri.017G082500	14	TCATCAACAGGTTCACAGCAA	CATACCCACAAGAGGGTTTCAA
	Potri.013G067500	15	GCTTTGGATCCTATGGCACA	CTTCAAGAAGGCGCGAAATG
	Potri.004G126700	16	GGCTGCCATAGCTTCATACC	GCCTAGCCATGTGGATATCAG
	Potri.010G123000	17	TGTGCAGGACTGAAGAGAAC	TGCAATCTTTGACCACCTGT
	Potri.010G149900	18	TGCTACTCATGGTGAGGGAC	ATTGCCCCATCGAGAATGAA
	Potri.008G122100	19	TTAGCAAGAGGTGCAGGACT	TGACCACCTGTTACCCCATT
	Potri.012G039200	20	GTCACGCGGTTCAGGTTTAT	TGCATAGAGTCTGTCCACCT
ERF	Potri.003G080600	1	TACAGAGGAGTCCGACGCA	AGAATCGCTTTCGACCCACG
	Potri.001G069300	2	AAATGACACCAACTGCCAGC	CCAAATCCTTGAACCCAGCC
	Potri.018G043900	3	CTGGGGCAAATGGGTGTCA	GTGAAGCTGGTCGAGGTAAGT
	Potri.006G253800	4	GCCAAGACCAATTTCCCAGC	GCCAGCTTTCTTTTGCCACA
	Potri.018G047300	5	ATTTCCCCGACAATCCACCG	CAGCTCCACCATCAGACGAC
	Potri.006G238600	6	TTCGGATGAGAAACTGGGGC	CAAGTGAAGCTGGACGAGGT
	Potri.012G108500	7	CATCTGGCGGGACCGAATTA	CACCAAATGGAACTGCAGGC
	Potri.006G218200	8	CTGACAACCCACCGGACATT	ATTTGTCCAGCCCCACAGTC
	Potri.T081300	9	TGGGCATGCCTATGACTTGG	TATTTGGACACACCGCGTGA
	Potri.016G018600	10	AGGGCCAAAATGCAGACCTT	TCATCGACCGTTGGCGTAAT
	Potri.005G223300	11	GAAAAGGAAGCCGCTCCGAA	GCCAAACTCGCACTCCATTT
	Potri.010G247200	12	TCGTCGTCTTCGCAACTTCA	ACAAGTCCGGTAACTCGCAG
	Potri.T050600	13	TATCAAGAGGAGCCACCCCA	CGGCCTCGGCAGTATCAAAT
	Potri.006G054500	14	ATTCATGCACAGCCTAGCGT	TCTCCTCATCTTTCTCTGCTGC
	Potri.014G076700	15	ACGAAGAGCCTGAAACACTG	TCAGCTACCCATCTTCCTGA
	Potri.017G087800	16	TGGGTGGCTGAGATTAAGGA	AAGTTGGAGAACAAGGCCAG
	Potri.008G091300	17	CGAAAACGATACCTTGGGGT	GTCCGAGTATTAGCTCCACG

	Potri.013G056700	18	AGGATGTGGCTAGGAACCTT	AGATTTCGAATCCGGGAAGC
	Potri.019G036100	19	TGCTTCGTGGATCAAACACT	TGGTGGGGTAGAGACTTCAG
	Potri.001G004700	20	AAGGCCTTCAGGAAAATGGG	TGGAGGGGACATGAGTGTTA
	Potri.003G220200	21	TAGACAAAGGCCTTCAGGGA	GTTCGAGTATTCGAGCCACG
AP2	Potri.T081300	1	GCAGCATTAAAGTACTGGGGA	GGTGTCTGGCTACACCTCTA
	Potri.010G181000	2	ACAATCAGCCTCAAGATTGGA	GCCACCAAGGAAGTTCTCAA
	Potri.003G185300	3	AAAGAGATGGAGGGTCATTCG	GGTAGAGGTACTTGTTGCCG
	Potri.010G247200	4	TGGACAGGAAGGTTTGAAGC	ATTCAAGGTTGTCTCTGACCC
	Potri.001G018400	5	TTCAAGCCAGTGATTCTGAGT	TACCACTAGCACTGCCCATA
	Potri.001G169500	6	CCGAAGTTCACTGTCAGCAA	AAGGTTTGCGCATTGTCATT
	Potri.003G064700	7	TGTCAGCAAGAAGATCACCAG	AGAAGAACGGCTTGTGAGAC
	Potri.006G179900	8	GCCTATGACTTAGCGGCATT	CCACGACTGCTTTTCCTTCT
WRKY	Potri.012G031700	1	AAGAAAGAGCATGCCGAGA	GAGAGGAAGCAAATTAGAGG
	Potri.003G138600	2	CAAAGAAGAGAAAGACCAC	TTCCATGAGCACAAGAGT
	Potri.014G096200	3	ACCAAGCCTCTCCTTCACCC	AATATCTCCTCTGTTGGCTTTGGT
	Potri.013G090300	4	AGTTACTTTAGGTGTTCTCG	AGAACTGATCACACTACTCG
	Potri.019G059300	5	TGCTCAACAGTTCGGCAATA	CAGGGATCGTCATCAAGTCG
	Potri.016G137900	6	TCGAACAAGCCAAGCAACTC	CATGATACCATCCATGTCCAG
	Potri.002G168700	7	TAGTCCTTGGAGTGAAGTC	GCATCTGTAGTATCCTCTTG
	Potri.014G096200	8	ACAGTAGTCCTAGGAGTG	GTAGTATTGCAATTGCAATG
	Potri.006G109100	9	TCCAACGATCCACAATAACC	TAAAACATCACCGCCGTCTC
	Potri.013G090400	10	CAGCCATGGAGACGTTGTTT	CTTCGAGGTCTTTGTGACGA
	Potri.012G031700	11	TACCGTGGAAGGCATACGTG	TCTGCGAAAGTTCGAGAGTAGT
	Potri.001G092900	12	CACATACCGAGGAACGCACT	TGTCGAAGTCCTCTGTATTAACCC
	Potri.003G138600	13	TCCATCACCATCATCACCAGAA	ATGCAGAGGGAAAAGAGAAGGG
	Potri.013G090300	14	GCAGAAGGGGATCTTGAATGC	ATGATTTGTGGAGCCTCAGGG
	Potri.019G059300	15	ACTACAGATGCACTCACCGT	TCATTTTCCAGTGAAGGCGATG
	Potri.014G096200	16	CCACGAAGCAAGTGCAAAGAT	GGTTTCTCTTGACGCTGCTG
	Potri.006G109100	17	ACCAACATTGCCAAGCAACC	TGATCAATCGGGTCCAAGACA
	Potri.013G090400	18	AGGATGATGCCGAAAGACGG	TGGTGGGTGCACCTGTAGTA

	Potri.001G092900	19	ACAGCAATCACAGCAAGCCC	GCCCTCACTCTTTGTGCATCC
	Potri.003G138600	20	AACAGCAGTCACAGCAAGCC	TGCAGAGGGAAAAGAGAAGGGA
	Potri.012G031700	21	ACCCGACCAAGAAAACAATCCAC	GGTTTGACAGTGGATGTTGAAGGG
	Potri.019G059300	22	TGTACCATTGCCCAGCCAAGA	TAATTCCCGGTGCTGGTGGT
18S ribosome RNA			CGAAGACGATCAGATACCGTCCTA	TTTCTCATAAGGTGCTGGCGGAGT

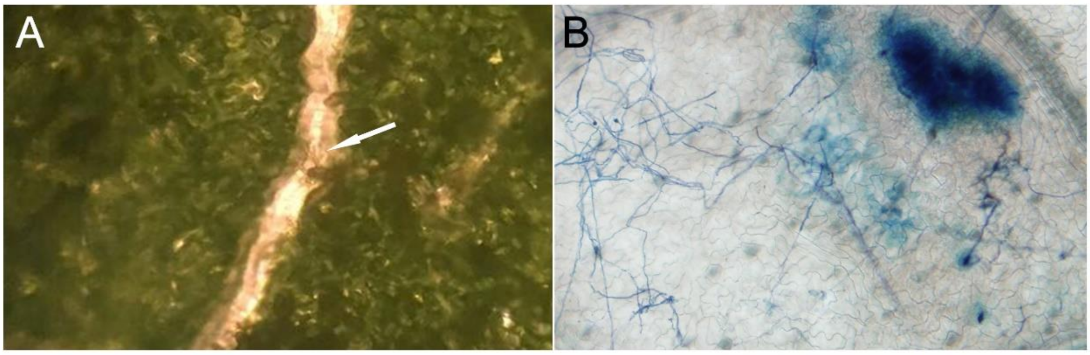

Figure S1. Symptoms of Rust+ 'Robusta' leaves at 12 hpi and 4 dpi. (A) 10× close-up of rust+ 'Robusta' leaf at 12 hpi. (B) 10× close-up of trypan blue staining on rust+ 'Robusta' leaf at 4 dpi.

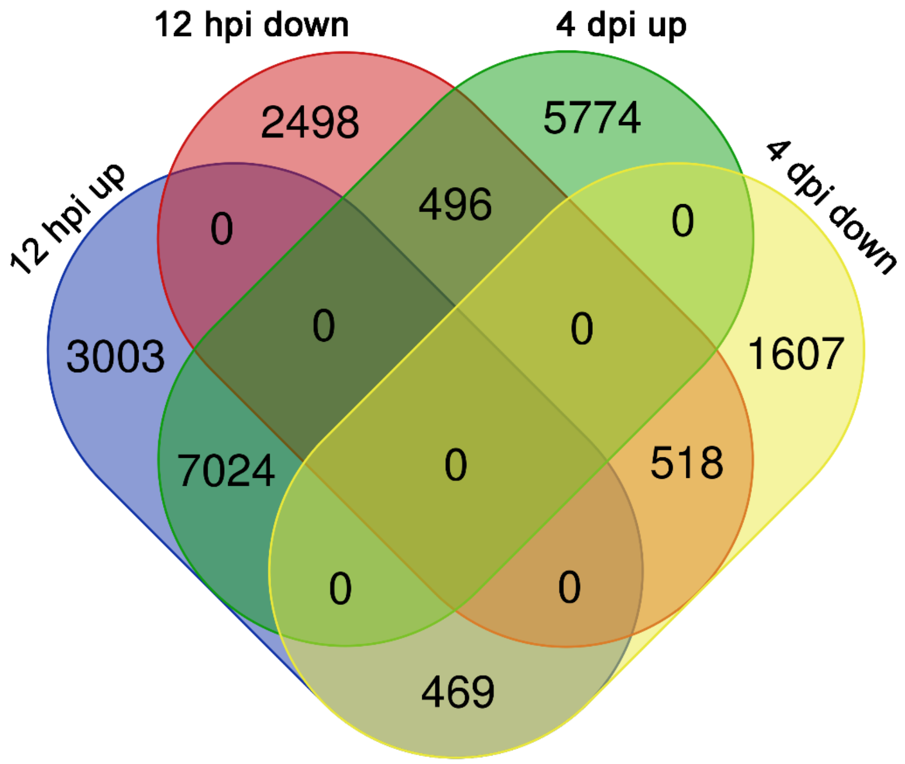

Figure S2. Differentially expressed genes analysis.

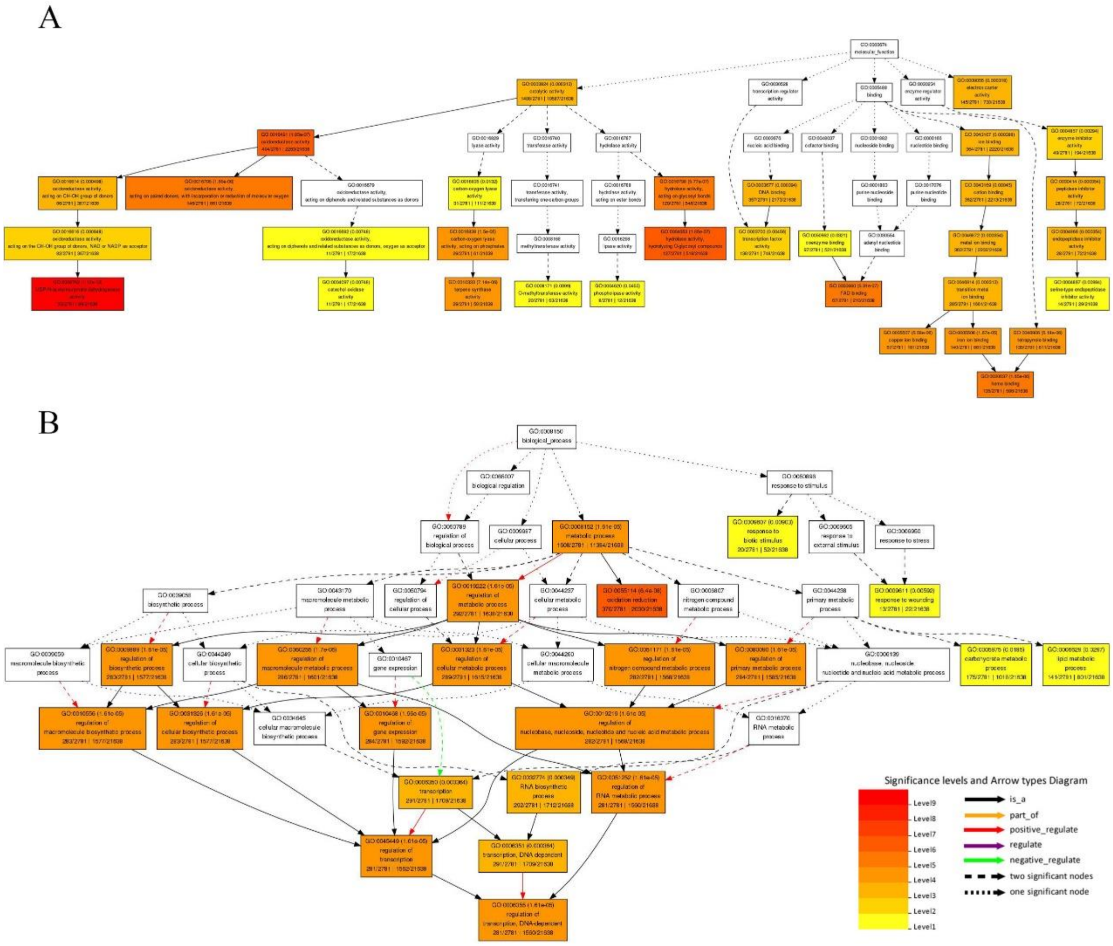

Figure S3. GO enrichment for all E4 responsive genes. (A) GO enrichment categorized as molecular function. (B) GO enrichment categorized as biological process.

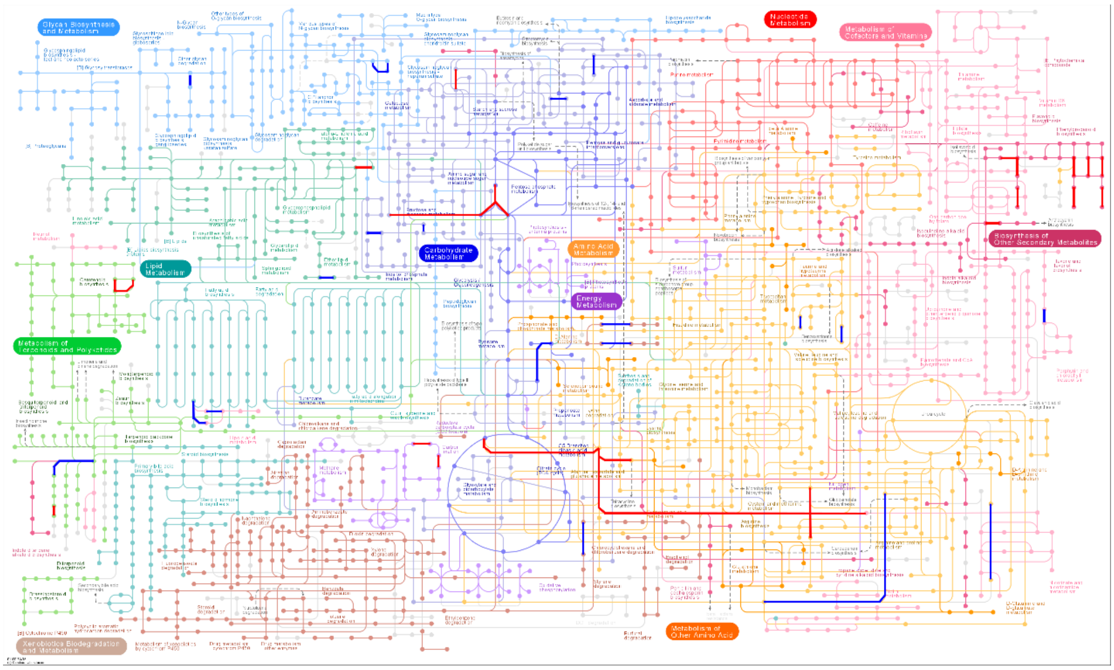

Figure S4. KEGG pathway and atlas.

Text S1 The annotation and expression of each gene

Clean data were got from the raw data (which contain the sequences and quality of reads) after adaptors cut, junk filter, and low copy filter according Illumina CASAVA v1.8+. Clean data were align with the mRNA of P. trichocarpa (ftp://ftp.jgi-psf.org) with Bowtie 1.1.2, and match data were named as mapped data. The annotation of each gene was get by the alignment of mapped data with the gene of P. trichocarpa. The expression of each gene was got by Weighted Algorithm as followed:

$$\text{Count(all)} = \sum_{i=0}^{n} \frac{CopyNum\ of\ reads(i)}{number\ of\ mapped\ mRNA(i)}$$

Text S2. Gene network analysis.

The bidirectional alignment of mapped data with the Arabidopsis interlogs were done online (http://www.uniprot.org/align/), then the Numbers of identified AGIs were got. Protein-protein interacting pairs (PPIs) was searched with SUBA3 (http://suba.plantenergy.uwa.edu.au/). A list of AGIs were entered into the searching box to rapidly view a specific subset of interacting proteins and the evidence for their location. Alternative splice information may be included. Visualization of the network was generated by Cytoscapev.3.3.0.
